# *Para*-N-Methylpyridinium Pyrenes: Impact of Positive Charge on ds-DNA/RNA and Protein Recognition, Photo-Induced Bioactivity, and Intracellular Localisation

**DOI:** 10.3390/pharmaceutics14112499

**Published:** 2022-11-17

**Authors:** Marta Košćak, Isabela Pehar, Ksenija Božinović, Goutam Kumar Kole, Sandra Sobočanec, Iva I. Podgorski, Marija Pinterić, Klaus Müller-Buschbaum, Dragomira Majhen, Ivo Piantanida, Todd B. Marder

**Affiliations:** 1 Division of Organic Chemistry and Biochemistry, Ruđer Bošković Institute, Bijenička Cesta 54, 10000 Zagreb, Croatia; 2 Division of Molecular Biology, Ruđer Bošković Institute, Bijenička Cesta 54, 10000 Zagreb, Croatia; 3 Department of Chemistry, College of Engineering and Technology, SRM Institute of Science and Technology, Kattankulathur 603203, Tamil Nadu, India; 4 Institut für Anorganische Chemie, Julius-Maximilians-Universität Würzburg, Am Hubland, 97074 Würzburg, Germany; 5 Division of Molecular Medicine, Ruđer Bošković Institute, Bijenička Cesta 54, 10000 Zagreb, Croatia; 6 Institut für Anorganische und Analytische Chemie, Justus-Liebig-Universität Gießen, Heinrich-Buff-Ring 17, 35392 Gießen, Germany

**Keywords:** N-methylpyridinium pyrene, DNA sensing, protein sensing, singlet oxygen, photodynamic therapy, fluorescence, theranostics

## Abstract

The 2- and 2,7- substituted *para*-N-methylpyridinium pyrene cations show high-affinity intercalation into ds-DNAs, whereas their non-methylated analogues interacted with ds-DNA/RNA only in the protonated form (at pH 5), but not at physiological conditions (pH 7). The fluorescence from non-methylated analogues was strongly dependent on the protonation of the pyridines; consequently, they act as fluorescence ratiometric probes for simultaneous detection of both ds-DNA and BSA at pH 5, relying on the ratio between intensities at 420 nm (BSA specific) and 520 nm (DNA specific), whereby exclusively ds-DNA sensing could be switched-off by adjustment to pH 7. Only methylated, permanently charged pyrenes show photoinduced cleavage of circular DNA, attributed to pyrene-mediated irradiation-induced production of singlet oxygen. Consequently, the moderate toxicity of these cations against human cell lines is strongly increased upon irradiation. Detailed studies revealed increased total ROS production in cells treated by the compounds studied, accompanied by cell swelling and augmentation of cellular complexity. The most photo-active 2-*para*-N-methylpyridinium pyrene showed significant localization at mitochondria, its photo-bioactivity likely due to mitochondrial DNA damage. Other derivatives were mostly non-selectively distributed between various cytoplasmic organelles, thus being less photoactive.

## 1. Introduction

Photodynamic therapy (PDT) is a treatment capable of inducing cell and tissue death by oxidative stress through light-induced activation of a nontoxic photosensitizer. The photosensitizer (PS) transfers energy from light to molecular oxygen, consequently generating, around the immediate PS location, various reactive oxygen species (ROS) [1]. Therefore, the biological responses to the photosensitizer are activated only in the particular areas of tissue that have been exposed to light [2], offering highly selective therapeutic targeting of well-defined and accessible malignant or potentially malignant conditions [2]. However, most photosensitizing (PS) agents are of high molecular weight, which hampers their administration to patients and results in long retention in blood, causing photodermatosis [3]. There are a few examples of low molecular weight PS agents, and we recently reported small molecules in PDT applications with some advantages regarding bioavailability and biolocalisation [4].

In this regard, condensed aromatics of low molecular weight, such as pyrene, with its favourable photosensitizability, could be efficient photosensitizers [5]. Although pyrenes are generally considered potentially carcinogenic, their substituted derivatives may serve as anticancer agents and chemotherapeutics [6,7]. For example, pyrene analogues caused increased ROS production in 3T3 fibroblast cells and HT29 colon cancer cells treated with (E)-pyrene oxime esters [8], while pyrene-induced cytotoxicity due to increased oxidative stress, i.e., ROS production, was observed in human HepG2 cells [9]. The fluorescence of pyrene derivatives allowed their application as fluorescent probes for various biomacromolecules, e.g., nucleic acids [10,11]. Some pyrene derivatives intercalate into DNA/RNA [12,13]; however, intercalative binding has limited biomedical selectivity and, therefore, some interesting applications of those pyrene derivatives rely on their interactions with DNA or RNA grooves [14,15] or use pyrene’s ability to form fluorescent excimers [16,17,18,19]. Thus, pyrene derivatives have already proven to be quite useful for diagnostic and sensing applications [20,21,22,23].

However, regarding PDT applications, one of the main disadvantages of most pyrene analogues is their non-favourable excitation wavelength (350–400 nm), far too short for efficient PDT due to limits to tissue permeability. Thus, for efficient tissue penetration, essential for PDT treatment, wavelengths > 600 nm are required [24]. However, recent developments in two-photon-absorption (TPA) allow the application of doubled excitation wavelengths for the efficient excitation of photosensitisers [25,26], thus allowing biomedical applicability even for pyrene analogues [27,28].

Since we developed the iridium-catalysed direct C-H borylation of pyrene at the 2-, and 2,7-positions as an efficient and selective route to this otherwise difficult-to-obtain substitution pattern [29,30], we have been exploring the synthesis, electronic, and optical properties, and various applications of 2-, and 2,7-substituted pyrenes [31,32,33,34,35,36,37,38,39,40,41,42,43,44], including a series of pyridyl-pyrenes [45].

**Scheme 1:**
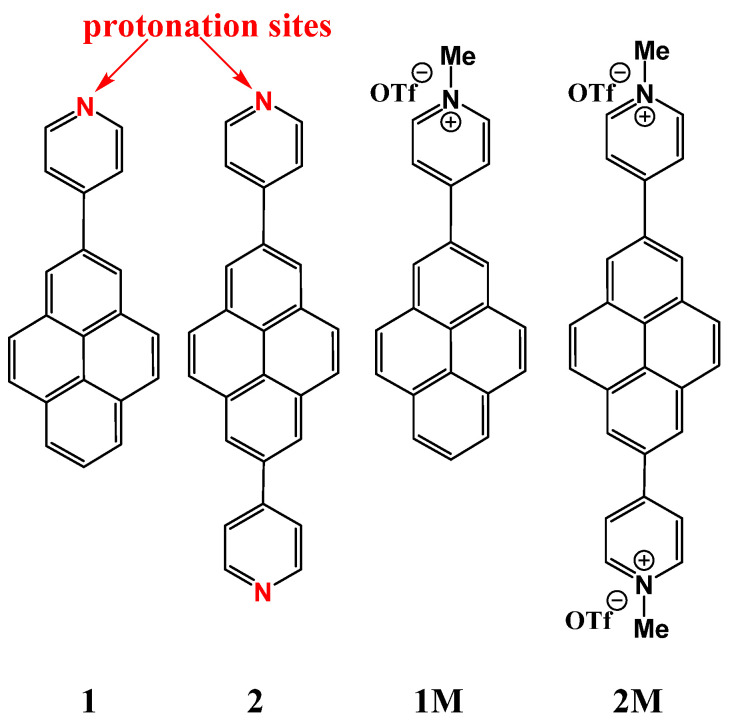
Structures of the compounds studied herein; note a permanent positive charge of **1M** and **2M** and protonation sites of **1** and **2**, allowing pH-control of positive charge.

Recently, we reported the mono- and bis-(N-methylpyridinium)-pyrenes **1M** and **2M** (Scheme 1) and analogous neutral compounds **1** and **2** [45], which have several properties highly applicable for PDT purposes [46]. For example, neutral compounds **1** and **2** revealed strongly bathochromically shifted absorption bands (375–475 nm), while preliminary studies showed that cationic **1M** and **2M** interact strongly with ct-DNA, the binding affinity depending on the number of positive charges.

These properties of **1M** and **2M** prompted us to study in detail their interactions with several of the most common ds-DNA or ds-RNA secondary structures, differing in steric and electronic properties of their binding sites for small molecules ( Supplementary Information Table S1). Even more intriguingly, our preliminary studies showed that neutral compounds **1** and **2** could be protonated (p*K_a_* ca. 5), and our previous studies of phenanthridine analogues [47] showed that only the protonated form (at pH 5) binds to ds-DNA and is biologically active. Such a pH-dependent property is the operational mode of several anticancer drugs, which thus accumulate in more acidic cancerous tissues [48,49,50,51]. Furthermore, we assessed the biological effects of these pyrene analogues (Scheme 1) on human cells in vitro by means of cytotoxicity, intracellular localization, and induction of ROS production in human cells, with and without light irradiation. As UV light irradiation increased the cell toxicity of the pyrene analogues, with the effect being the most pronounced for **1M**, our additional studies suggested that the particularly increased cell toxicity of **1M** upon UV irradiation can be attributed to increased production of ROS inside mitochondria and consequent DNA damage. Data presented here provide valuable information on the potential mechanisms involved in the cytotoxicity induced by mono- and bis-(N-methylpyridinium)-pyrenes, suggesting **1M** as a potential photosensitizer for photodynamic therapy.

## 2. Materials and Methods

The synthesis and detailed spectrophotometric characterisation of compounds **1**, **2**, **1M**, and **2M** were reported previously [46].

### Study of Interactions with ds-DNA and ds-RNA

Experiments were conducted in an aqueous buffer solution (pH = 7.0 or pH= 5.0, *I* = 0.05 M, sodium cacodylate buffer). For the UV-Vis spectra were used Varian Cary 100 Bio spectrometer, for fluorescence spectra we used the Varian Cary Eclipse fluorimeter and for CD spectra we used the JASCO J815 spectropolarimeter, all conducted at 25.0 °C using appropriate quartz cuvettes (path length: 1 cm).

Polynucleotides were purchased as noted: poly dAdT-poly dAdT, poly dGdC-poly dGdC, poly A-poly U (ds-RNA), (Sigma-Aldrich, Schnelldorf, Germany), calf thymus (ct)-DNA (Sigma-Aldrich, Schnelldorf, Germany) and dissolved in sodium cacodylate buffer, *I* = 0.05 M, pH = 7.0. The ct-DNA was sonicated and filtered through a 0.45 µm filter to obtain mostly short (ca. 100 base pairs) rod-like B-helical DNA fragments. The concentration of DNA or RNA was determined spectroscopically as the *c*(nucleobase) using producer-provided molar extinction coefficients.

Circular dichroism (CD) spectra were collected with a scanning speed of 200 nm/min and with three accumulations averaging. A buffer background was subtracted from each spectrum. CD experiments were performed by titrating the compound stock solution into the polynucleotide solution (*c* = 2 × 10^−5^ M).

Thermal denaturation of ds-DNA, ds-RNA and their complexes with studied compounds was performed [52] by monitoring the absorption change at 260 nm as a function of temperature. *T_m_* values are the midpoints of the transition curves determined from the maximum of the first derivative and checked graphically by the tangent method. The ∆*T_m_* values were calculated by subtracting the *T_m_* of the free nucleic acid from the *T_m_* of the complex. Every ∆*T_m_* value here reported was the average of at least two measurements. The error in ∆*T_m_* is ± 0.5 °C.

***Cells.*** Commercially available from American Type Culture Collection (ATCC), human epithelial lung adenocarcinoma A549 (ATCC^®^ CCL-185™) and human normal lung fibroblast WI-38 (ATCC^®^ CCL-75™) were grown according to supplier’s conditions, namely in Dulbecco Modified Eagle’s Medium (DMEM, Sigma Aldrich, St. Louis, MO, USA) supplemented with 10% of fetal bovine serum (FBS, Sigma Aldrich, USA). Cells were incubated in the cell incubator (Thermo Fischer Scientific, Waltham, MA, USA) at 37 °C and 5% CO_2_ in a humidified atmosphere.

***Cell viability assay.*** In order to obtain a 10 mM stock solution chemical compounds (**2M**, **1**, and **1M**) were sterilely diluted in an appropriate volume of dimethyl sulfoxide solution (DMSO, Gram-Mol, Zagreb, Croatia). Solutions were kept in the dark and stored at 4 °C. The cytotoxic effects of each compound were assayed on A549 and WI-38 cells. Working solutions, prepared by diluting DMSO stock solutions in DMEM, taking care that DMSO content does not exceed 0.1%, and tested by MTT test [53]. Briefly, cells were seeded in 96-well tissue culture plates and 24 h later treated with **2M**, **1**, or **1M** in the concentration range of 10–0.1 μM. Cells treated with the same dilutions of DMSO represented the control, while cells treated only with DMEM (10% FBS) represented the negative control. After 72 h, the media was removed, MTT solution was added into each well, and the plate was incubated (37 °C, 5% CO_2_) for 3 h. The resulting MTT-formazan products were dissolved using DMSO, and their absorbance at 600 nm was measured using a microplate reader.

For determining the effect of UV exposure, cells were seeded on two plates and treated with working solutions of the respective compounds as previously described. Plates were incubated (37 °C, 5% CO_2_) for 90 min, allowing the compounds to enter the cells. Then, one plate was exposed to UV light (Luzchem reactor, 350 nm, 8 lamps, in total 8 W, dose 50.6 mWm^−2^; ~18 cm lamp to cell-plate) for 5 min, 3 days in a row at the same time each day, while the other plate was left in the cell incubator in the dark and served as a control.

***Live cell imaging by confocal microscopy.***

For the live imaging A549 cells were seeded in Ibidi imaging cell chambers (Ibidi^®^, Gräfelfing, Germany) and the next day treated with a 10 μM solution of the respective compound. Cells were left in the cell incubator for 90 min to allow the compound to enter the cells. After incubation, the medium was replaced with 500 μL of fresh medium. The influence of the compounds on A549 cells before and after photoactivation (exc = 405 nm, em = 450–550 nm) was visualised using a Leica SP8 X confocal microscope (Leica Microsystems, Wetzlar, Germany).

To analyse localisation at mitochondria after incubation with the respective compounds, cells were rinsed and incubated with a 100 nM MitoTracker Deep Red solution (Invitrogen, Molecular Probes, Waltham, MA, USA). MitoTracker dye was added to cells for 20 min at 37 °C, after which the medium was replaced with 500 μL of fresh DMEM and cells were immediately observed with a Leica SP8 X confocal microscope and the degree of colocalisation was assessed via the Pearson correlation coefficient, r.

***Immunofluorescence.*** A549 cells were seeded on coverslips in 24-well tissue culture plates (3 × 10^4^ cells/well), which were incubated in FBS for 2 h (37 °C, 5% CO_2_) prior to seeding; 48 h later, the cells were treated with the respective compound for 90 min (37 °C, 5% CO_2_), allowing the compound to enter to cells. After the treatment, cells were washed with PBS and incubated in 2% paraformaldehyde in PBS for 12 min at RT and 0.5% Triton X-100/PBS for 2 min. After fixation and permeabilisation, samples were washed with PBS, blocked with 3% bovine serum albumin in PBS for 30 min at RT and incubated with primary antibodies specific for EEA1 (#2411, Cell Signaling, Danvers, MA, SAD), LAMP1 (ab24170, Abcam, Cambridge, UK) or GM130 (#1248, Cell Signaling, Danvers, MA, SAD) respectively, in 5% BSA in PBS for 1 h, at RT. Samples were subsequently washed with PBS and then incubated with the appropriate Alexa Fluor-conjugated secondary antibody in 5% BSA in PBS for 1 h, at RT and in the dark. Coverslips were mounted in mounting medium (Fluoromount G, Southern Biotech, Birmingham, AL, USA). Confocal microscopy analyses were performed using the Leica TCS SP8 X microscope with a 63x objective. Images were processed with the Leica Application Suite X (LAS X, Leica, Wetzlar, Germany) software platform. Colocalisation was assessed by the Pearson coefficient. Analysis was conducted using ImageJ software and the appropriate JACoP plugin [30].

***Detection of total ROS.*** For detecting total ROS, A549 cells were seeded in 12-well tissue culture plates and 24 h after were treated with a 10 μM working solution of the respective compound and incubated for 90 min (37 °C, 5% CO_2_), allowing the compound to enter to cells. The same dilution of DMSO served as a control, while H2O2 (0.002%) was used as a positive control. One plate was irradiated with UV light (350 nm) for 5 min, and was analysed immediately after UV exposure. The plate that was not irradiated with UV light served as the control, and was incubated in the cell incubator (37°C, 5% CO_2_) during the whole experiment. Subsequently, cells were washed with PBS, trypsinised, resuspended with cold PBS supplemented with 10% FBS and centrifuged for 5 min (RT, 1100 g). Cell pellets were resuspended and washed again, but this time with cold PBS supplemented with 1% FBS and centrifuged for 5 min (RT, 1100 g). After the second washing, cell pellets were resuspended in 5 μM 2′,7′–dichloro-fluorescin diacetate (H2DCFDA, Sigma, USA) which is converted to dichlorofluorescein (DCF) in contact with ROS. Unstained cells were resuspended in PBS supplemented with 1% FBS. Cells were then analysed by flow cytometry using FACSCalibur (BD Biosciences, San Jose, CA, USA).

For measuring mitochondrial ROS, A549 cells were treated with 1 μM of the respective compound and incubated for 90 min (37 °C, 5% CO_2_), allowing the compound to enter to cells. Mitochondrial superoxide production was assessed with MitoSOX Red reagent (Thermo Fisher Scientific, Waltham, MA). The cells were washed with PBS and trypsinized, and then kept on ice unless otherwise specified. Cells were distributed into small tubes (3 × 10^5^ cells per tube) and resuspended in 5 µM MitoSOX solution prepared in PBS. The cells were incubated on a thermomixer for 30 min at 37 °C, with shaking (300 rpm), in the dark, followed by two washes with PBS. Then, cells dyed with MitoSOX were re-suspended in 400 µL of PBS and distributed into black 96-well plates, 120 µL per well (in triplicate). A Tecan Infinite 200 fluorescence microplate reader with 485/520 and 520/590 nm excitation/emission wavelengths was used to measure MitoSOX fluorescence.

***Plasmid electrophoresis.*** For assessing the photoinduced DNA nuclease activity of compounds **2M**, **1** and **1M**, 1 μg of pUC19 plasmid DNA (2686 pb) was incubated with a 100 μM solution of the respective compound, and exposed to UV light (using a xenon arc lamp, Oriel, Stratford, CT, USA) for 15 and 45 s. After the treatment plasmid samples were run on 0.5% agarose gel, and stained with Midori Green DNA binding dye (Nippon Genetics, Düren, Germany). Non-irradiated samples containing compounds and plasmid DNA served as a control for assessing the photoinduced nuclease activity of the compound. Plasmid without the compound was irradiated with UV light under the same conditions to determine whether UV light itself induces changes in plasmid DNA forms (linear and supercoiled form). After gel electrophoresis, plasmid DNA forms were visualised on a UVITEC Imager (Cleaver Scientific, Rugby, UK).

***Statistical analysis.*** Each experiment was repeated at least three times unless otherwise noted. The data were analysed by the unpaired Student’s *t*-test and expressed as means ± standard deviation (SD). Data were considered statistically significant at a *p*-value < 0.05.

## 3. Results

### 3.1. Protonation Properties of Neutral Analogues ***1*** and ***2***

**Figure 1:**
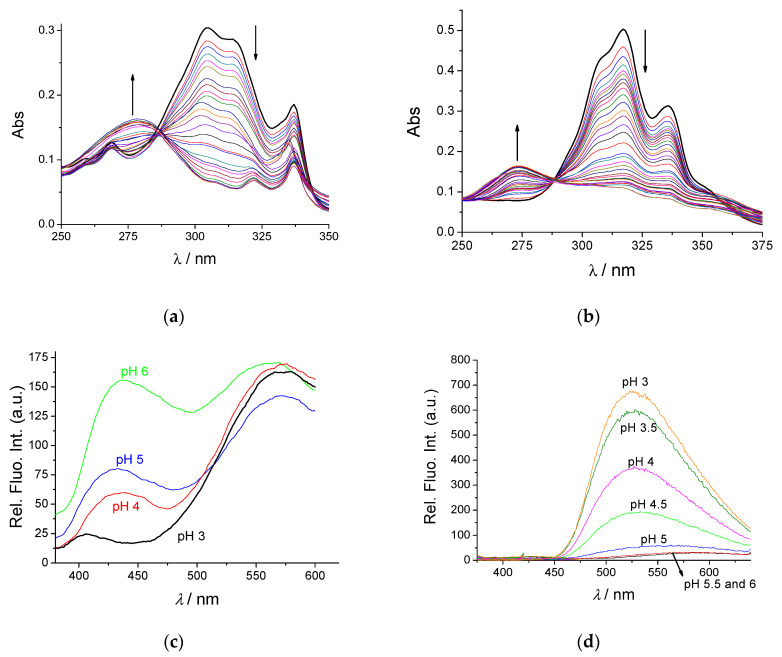
(**a**) Dependence of the UV/vis spectrum on pH (*c*(**1** or **2**) = 2.0 × 10^−5^ M) of (**a**) **1** (p*K_a_*~5); (**b**) **2** (p*K_a_* < 4). Dependence of the fluorescence spectrum on pH (*c*(**1** or **2**) = 1.0 × 10^−6^ M) of (**c**) **1** (λ_exc_ = 340 nm); (**d**) **2** (λ_exc_ = 332 nm). Performed in water by adjusting pH to 3 with HCl and subsequent additions of NaOH solution, simultaneously measuring pH and collecting UV/vis spectra.

Neutral analogues **1** and **2** are poorly soluble in water; therefore, we dissolved them at acidic conditions (0.001 M HCl in miliQ water), at *c* = 2.0 × 10^−5^ M and subsequently added copious amounts of NaOH solution, simultaneously measuring the pH and collecting UV/vis spectra (Figure 1a,b). Analysis of the results obtained (Supplementary Information, Figures S1 and S2) yielded a p*K_a_*~5 for the mono-protonated derivative **1** and colloidisation was observed for **2** in the process of adding NaOH, allowing only an estimation of a p*K_a_* < 4.

We also studied the emission from neutral **1** and **2** in water at various pH values (Figure 1c,d and Supplementary Information, Figure S3). Comparison of the results with those previously obtained for **1M** and **2M** as well as **1** and **2** (in MeCN) [46], revealed that the emission from **1** in its completely protonated form (pH 3) is characterised by a single emission maximum at 560 nm, agreeing with emission spectra of **1M**, whereas in the process of partial deprotonation of **1** (pH >4) a new emission maximum appeared at ca. 400–430 nm, agreeing nicely with the emission of **1** in MeCN) [46]. Analogously, the emission from fully protonated **2** (pH 3) agreed with the emission from **2M**; however, deprotonation of **2** (pH > 4) only decreased the emission at 540 nm, but showed no emission at 400–450 nm (as observed for **2** in MeCN [46]).

A pH of 5 is still biologically relevant for more acidic cancerous tissues and pH-controlled DNA binding [47,48,49,50,51]; thus, in further experiments, we studied the interactions of cationic **1M** and **2M** at pH 7 and neutral **1, 2** at pH 5 (at which **1** or **2** are at least partially protonated and could have electrostatic interactions with DNA/RNA).

### 3.2. Non-Covalent Interactions of ***1***, ***2***, ***1M*** and ***2M*** with Various ds-DNA, ds-RNA, and BSA

Preliminary screening conducted at pH 7 revealed strong interactions of permanently charged **1M** and **2M** with ct-DNA, [46] while neutral **1** or **2** did not show any interaction (data not shown). Thus, we tested **1, 2** in detail only at pH 5, at which they showed measurable interactions with DNA/RNA.

#### 3.2.1. Thermal Denaturation of ds-DNA/RNA

Thermally-induced dissociation of the ds-polynucleotides occurs at a well-defined temperature (*T*_m_ value), thus being used for the characterisation of various ds-DNA or ds-RNA-related processes. For example, noncovalent binding of small molecules to ds-polynucleotides usually increases the thermal stability of the ds-helices, and this increase (∆*T*_m_ value) can be correlated with the various binding modes; however, this requires confirmation by an independent method [52]. For example, most pyrene analogues, by intercalating into ds-DNA, cause stabilisation for ∆*T*_m_ > 5 °C due to aromatic stacking interactions with DNA base pairs, whereas the binding of pyrenes within the polynucleotide groove, usually driven by hydrophobic effects, should have a negligible stabilising outcome [14].

Thus, we performed thermal denaturation experiments with several DNAs or RNA, whereby added **1**, **1M,** or **2M** generally stabilised the polynucleotides against denaturation (Table 1).

The addition of **2M** induced strong stabilisation of both ct-DNA and poly dAdT-poly dAdT (Table 1) indicating **2M** intercalation between base pairs, typical for pyrenes due to their planar structure. For ratios r = 0.2–0.3, there is no significant difference in polynucleotide stabilisation, suggesting saturation of the binding sites at the ratio r = 0.2. The stabilisation of ds-RNA, upon the addition of **2M**, was weaker than with ds-DNA. The addition of **1M** caused moderate and similar stabilisation of ds-DNA and ds-RNA, while non-methylated analogues **1** and **2** weakly stabilised ds-DNA only at pH 5, at which they are partially protonated, thus stressing the importance of positive charge for interaction with ds-DNA.

#### 3.2.2. Spectrophotometric Titrations of Pyrene Analogues with DNA, RNA, BSA

As the pyrene analogues studied are strong chromophores and fluorophores, we examined their interactions with DNA/RNA and bovine serum albumin (BSA) by monitoring changes in their UV/vis absorption and fluorescence spectra.

**Figure 2:**
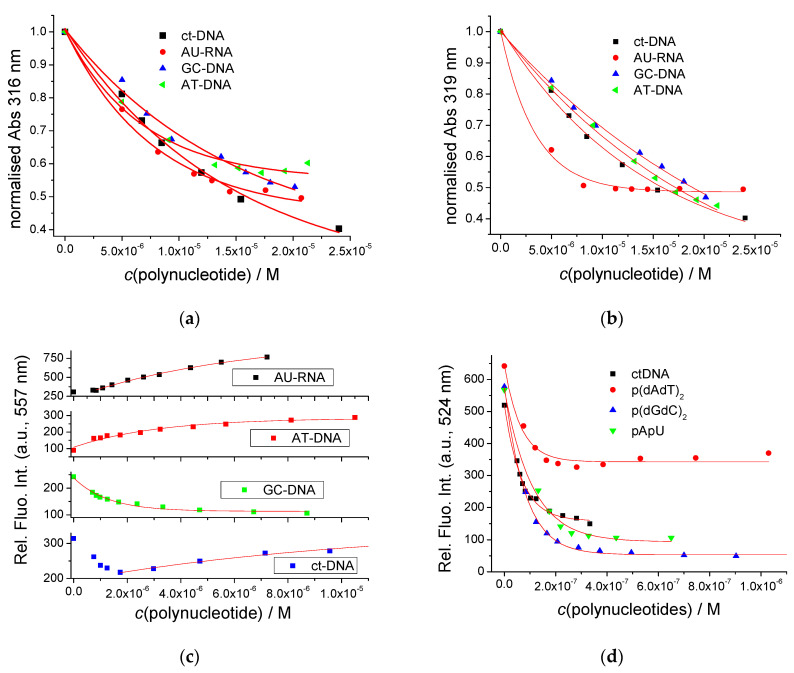
Changes at UV/vis maxima of (**a**) **1M** and (**b**) **2M** (*c* (dye) = 5 × 10^−6^ M) upon addition of polynucleotides; the Y-axis was normalised to starting absorbance value of a free dye. Changes at fluorescence maxima of (**c**) **1M** and (**d**) **2M** (*c* (dye) = 5 × 10^−8^ M) upon addition of polynucleotides. All titrations were conducted at pH 7.0, sodium cacodylate buffer*, I* = 50 mM.

In general, the addition of any ds-DNA or ds-RNA to **1M** or **2M** resulted in a strong hypochromic and bathochromic effect in **1M** or **2M** UV/vis spectra (Figure 2a,b;  Supplementary Information Figures S4–S11), characteristic of the strong engagement of the pyrene chromophore in aromatic stacking interactions [20]. Mono-methylated **1M** UV/vis changes showed no selectivity towards any of the polynucleotides studied (Figure 2a), while bis-methylated **2M** shows an increased affinity towards AU-RNA in comparison to all ds-DNAs (Figure 2b). However, it should be stressed that, for some UV/vis titrations, a deviation from the isosbestic point was observed at an excess of the dye over c(DNA/RNA binding sites), which suggests aggregation of the dye along polynucleotide, which would influence the value of binding constant.

At concentrations necessary for accurate UV/vis titrations (*c* = 5 × 10^−6^ M), non-methylated **1** or **2** precipitated upon DNA/RNA addition.

Fortunately, strong emission from the compounds studied allowed fluorimetric titrations to be performed at much lower concentrations of the dyes (*c* = 5 × 10^−8^ M), thus preventing precipitation of non-methylated **1** or **2**, and allowing for the collection of numerous data points at excess of DNA/RNA binding sites over *c*(dye). The addition of DNA/RNA resulted in strong emission changes of **1M** or **2M**; however, mono-methylated **1M** showed an emission increase only for AT-DNA and AU-RNA (Figure 2c), while GC-DNA quenched its emission. Such base-pair-dependent emission selectivity was observed for some 4,9-diazapyrenium compounds [54] and proflavine [55], attributed to the difference in the redox potential of nucleobases. Intriguingly, mixed sequence ct-DNA caused emission quenching of **1M** at an excess of dye over ct-DNA (r > 0.1), and such quenching could be attributed to aggregation of **1M** along DNA, while at an excess of ct-DNA over dye (r << 0.1), emission increased. The emission of **2M** was indiscriminately quenched by all ds-DNA/RNA (Figure 2d).

As the pharmacokinetic properties of small molecules often depend on a binding affinity toward transport proteins (such as serum albumin), we performed fluorimetric titrations with BSA. The emission of both **1M** and **2M** (Supplementary Information, Figures S15 and S16), was strongly quenched, with compound **2M** showing a lower affinity in comparison to ds-DNA/RNA, while the affinity of **1M** was similar to that towards ds-DNA/RNA (Table 2).

**Figure 3:**
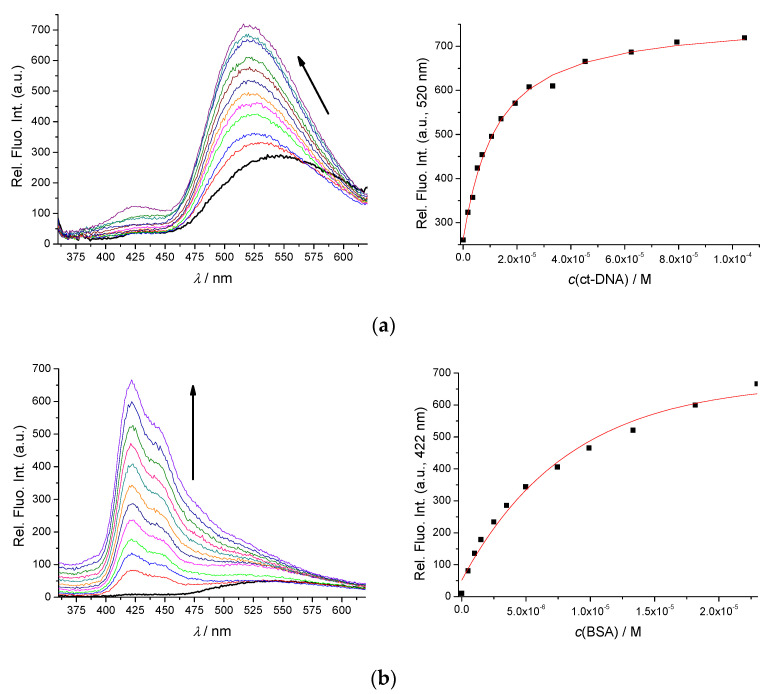
Changes in the fluorescence spectrum of **2** (*c* = 5 × 10^−7^ M, λ_exc_ = 319 nm, pH 5.0, sodium cacodylate buffer, *I* = 0.05 M) upon titration with (**a**) ct-DNA; (**b**) BSA.

Upon the addition of ct-DNA at pH 7, the fluorescence of non-methylated compounds **1** and **2** changed only negligibly, whereas, at pH 5, the emission of both compounds strongly increased (Figure 3a, Supplementary Information Figure S12). Most intriguingly, at both pH 7 and pH 5, fluorescence from **1** and **2** strongly increased upon the addition of BSA, accompanied by more than a 100 nm hypsochromically shifted emission maximum (Figure 3b, Supplementary Information Figures S13 and S14).

Comparison of the results obtained (Figure 3) with previous studies of **2** and its protonated analogue **2H** [46] revealed strong similarities between the emission spectrum of the **2**/DNA complex and the emission spectrum of protonated **2H**, as well as between the **2**/BSA complex and the non-protonated form of **2**. The analogous similarity was observed for **1**.

Thus, **1** and **2** bind to ds-DNA only in their protonated forms (and therefore interact only at pH 5), at variance to BSA, which has highly hydrophobic binding sites to which only neutral **1** and **2** are bound similarly at both pH 5 and pH 7.

Processing of fluorimetric titrations by means of the Scatchard equation [56,57] yielded binding constants (Table 2). Bis-methylated **2M** revealed a much higher affinity toward all DNA/RNA in comparison to **1M**, agreeing well with higher thermal stabilisation (Table 1). Non-methylated **2**, due to only partial protonation at pH 5, showed a much lower affinity than **2M**. Affinities of **1** and **2** toward BSA were the same at both pH values, supporting the conclusion that neutral forms of **1** and **2** were bound.

Thus, **1** and **2** can be considered as ratiometric probes for the simultaneous detection of both ds-DNA and BSA at pH 5, relying on the ratio between the intensities at 420 nm (BSA specific) and 520 nm (DNA specific), respectively. Even more useful is the possibility to switch-off ds-DNA sensing by adjustment to pH 7, at which only BSA would yield a measurable emission change.

#### 3.2.3. Circular Dichroism (CD) Experiments

The circular dichroism (CD) spectropolarimetrycan give additional information about the structural properties of the small molecule/polynucleotide complexes. For instance, achiral small molecules, such as **1**, **2**, **1M** or **2M**, can acquire an induced CD spectrum (ICD) upon binding to polynucleotides, directly dependent on a dominant mode of interaction [58,59].

**Figure 4:**
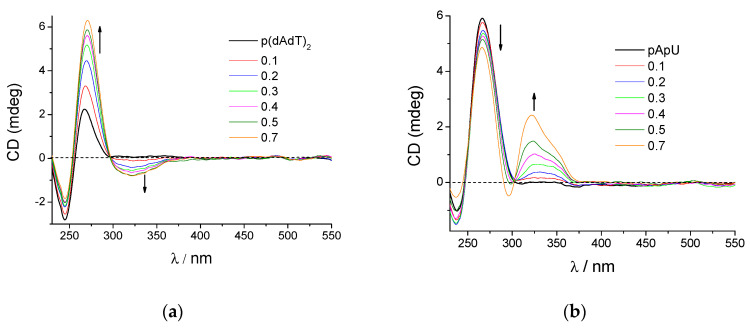
CD titration of (**a**) poly dAdT-poly dAdT, (**b**) pApU (c = 2 × 10^−5^ M) with **1M** at molar ratios r = [compound]/[polynucleotide] (pH 7.0, sodium cacodylate buffer, *I* = 0.05 M).

**Figure 5:**
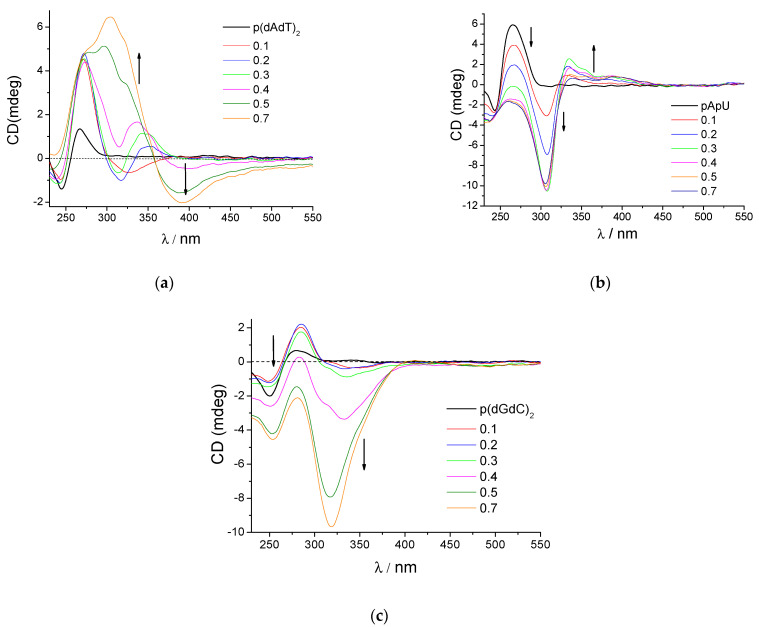
CD titration of (**a**) p(dAdT)_2_, (**b**) poly A–poly U, (**c**) p(dGdC)_2_ (*c* = 2 × 10^−5^ M) with **2M** at molar ratios r = [compound]/[polynucleotide] (pH 7.0, sodium cacodylate buffer, *I* = 0.05 M).

The addition of both **1M** and **2M** induced pronounced changes in the CD spectra of ds-DNAs and ds-RNA (Figure 4 and Figure 5), which were strongly dependent on the secondary structure of the ds-polynucleotide (Supplementary Information, Table S1) and on the ratio r = [compound]/[polynucleotide].

For AT-DNA, at a low ratio (r ≤ 0.1), weak negative ICD bands at λ = 300−350 nm were observed for **1M** and **2M**, indicative of intercalation between base pairs [56,57], which agrees well with the bathochromic and hypochromic changes in UV/vis titrations, high binding constants, and pronounced thermal denaturation stabilisation. The monomethylated **1M/**AT-DNA complex was characterised by an isoelliptic point at 297 nm (Figure 4a), supporting the formation of only one dominant type of complex. In contrast, further additions of **2M** (r > 0.2) resulted in a strong negative ICD pattern, suggesting aggregation of pyrene molecules along ds-DNA [59].

The effect of **1M** or **2M** addition to AU-RNA was substantially different regarding the ICD band range at λ = 300−400 nm; at all ratios r, **1M** caused a systematic increase of the positive ICD band with isoelliptic point at 300 nm (Figure 4b), which is characteristic of RNA major groove binding of a single molecule [59], while the addition of **2M** resulted in very strong bisignate ICD bands with coupled negative-positive ICD bands in 280–400 nm range (Figure 5b) ascribed to the formation of pyrene-dimers or higher aggregates within the RNA major groove [59].

At an excess of GC-DNA over **2M** (Figure 5c, ratio r < 0.2), a weak negative ICD band at 310-340 nm appeared, typical of intercalative binding [58,59]. However, only **2M** induced an exceptionally strong negative ICD band at an excess of **2M** over GC-DNA (Figure 5c, r > 0.3), its minimum changing with each addition of **2M**, which could be attributed to the aggregation of pyrene-units in the strongly chiral structure.

CD spectra of ds-DNA changed only slightly upon the addition of non-methylated **1** or **2** at pH 5 (Supplementary Information, Figure S24), and no measurable ICD bands were observed, suggesting binding within the minor groove of DNA.

#### 3.2.4. ***2M*** and ***1M*** Induce Plasmid DNA Cleavage

**Figure 6:**
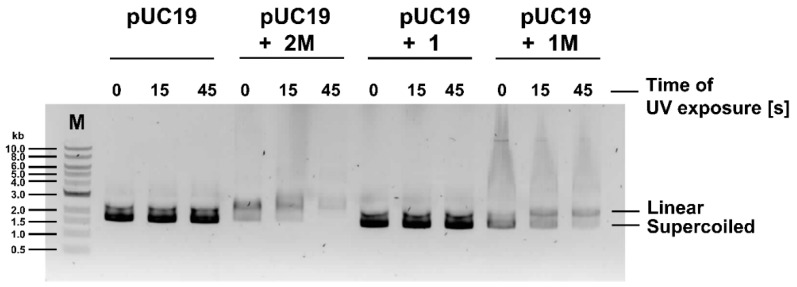
Representative agarose gel of pUC19 plasmid DNA incubated with **2M**, **1**, or **1M**, with or without exposure to UV light (15 or 45 s). M–GelPilot DNA Molecular Weight Marker 1kb. Representative data from two independent experiments that yielded similar results are shown.

Due to its photophysical properties, allowing the photo-induced generation of singlet oxygen [46], as well as strong binding to ds-DNA, (*vide supra*) the pyrenes studied, can cause DNA cleavage upon UV irradiation [20]. In order to determine whether **2M**, **1**, and **1M** possess DNA photo cleavage properties, we assessed plasmid DNA cleavage in the presence of **2M**, **1**, and **1M** with or without UV irradiation (Figure 6).

As shown in Figure 6, both **2M** and **1M** exhibited strong photo-induced nuclease activity. Furthermore, dicationic **2M** caused the unwinding of supercoiled DNA even without UV irradiation, which is in line with its capacity to intercalate into DNA, while monocationic **1M** induced linearisation of plasmid DNA only after UV exposure. Compound **1M** also caused nicking of the plasmid DNA, which was more pronounced under non-irradiated conditions. Non-DNA-intercalating neutral **1** did not show any influence on DNA plasmid forms, likely because compound **1** is not protonated at pH 7 and does not bind to DNA. As UV irradiation had no influence on the DNA plasmid forms itself, it is evident that linearisation of plasmid DNA was induced solely by the photo-activated compounds **2M** and **1M**.

## 4. Biological Activity of 1, 2, 1M, and 2M

### 4.1. UV Light Irradiation Increases the Cell Toxicity of ***2M***, ***1*** or ***1M***

**Figure 7:**
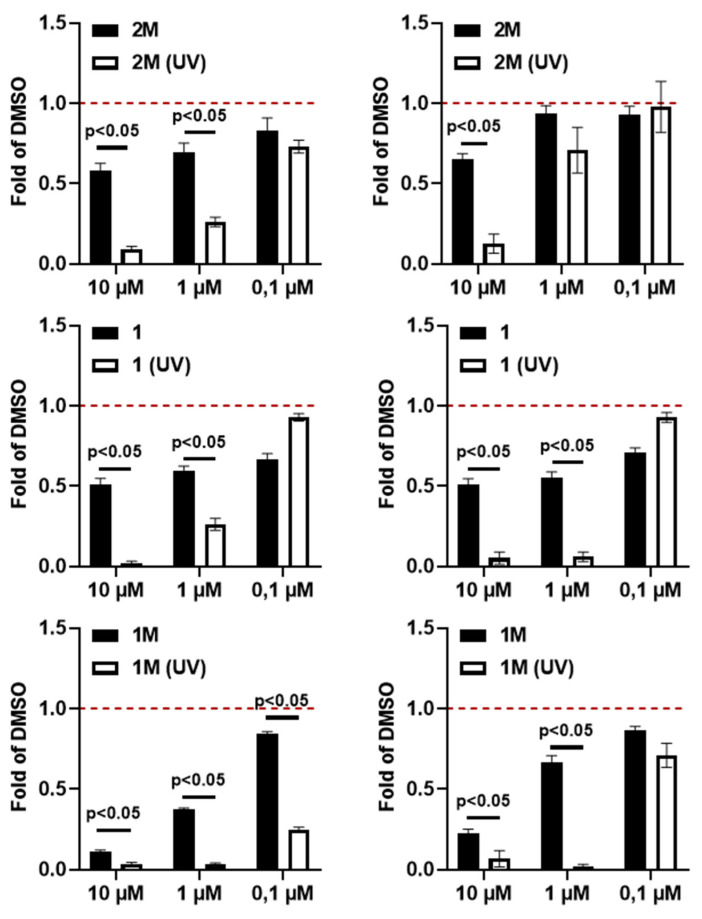
Cell toxicity of **1**, **1M**, and **2M** in A549 and WI-38 cell lines, with or without exposure to UV light. Data are presented as a fold of control cell treated with an amount of DMSO corresponding to an added aliquot of dye DMSO stock solution (<0.1% DMSO in medium), with ± SD resulting from the four replicates. Representative data from two independent experiments which yielded similar results are shown, with good statistical significance values *p* < 0.05.

The cytotoxic effect of **2M**, **1**, and **1M** on human lung carcinoma (A549) and normal lung (WI-38) cell lines were treated with different concentrations of **2M**, **1**, and **1M** (10 μM, 1 μM and 0.1 μM) and cell survival was assessed by the MTT assay (Figure 7).

All three compounds, **2M**, **1**, and **1M**, at 10 μM and 1 μM concentrations caused considerable cytotoxic effects in the A549 cell line. Under the same conditions, the WI-38 cell line was somewhat more resilient, particularly to cationic **1M** and **2M**. The cytotoxic effect of **2M**, **1**, and **1M** was further increased by exposing treated cells to UV light. A significant decrease in cell survival was observed at higher concentrations of the compounds (10 µM and 1 µM) under UV light irradiation, the effect being more pronounced in tumour A549 cells compared to the normal WI-38 cell line. In addition, for the A549 cell line, a comparison of the IC50 values obtained for **2M** and **1** revealed four times lower toxicity (8 µM vs. 2 µM) under non-irradiated conditions compared to the UV-irradiated experiment, while for **1M** difference was even larger, UV-irradiation causing 15-times stronger IC50 values (1.5 µM vs. 0.1 µM). For the normal WI-38 cell line treated with our compounds, the effect of UV irradiation was similar to that observed for the A549 cell line. Thus, **1M** exhibits the strongest photo-induced cytotoxicity, quite significant even at 0.1 µM concentrations, and comparable to many established antitumour drugs (e.g., doxorubicin). Compound **2** showed no cytotoxic effect (Supplementary Information, Figure S25) but, due to its low solubility, it was not included in further biological characterisation. Due to its lower p*K_a_* and tendency to precipitate, we did not study the biological action of compound **2** further.

### 4.2. UV Light Irradiation Increases ROS Production in Cells Exposed to ***2M***, ***1***, or ***1M***

**Figure 8:**
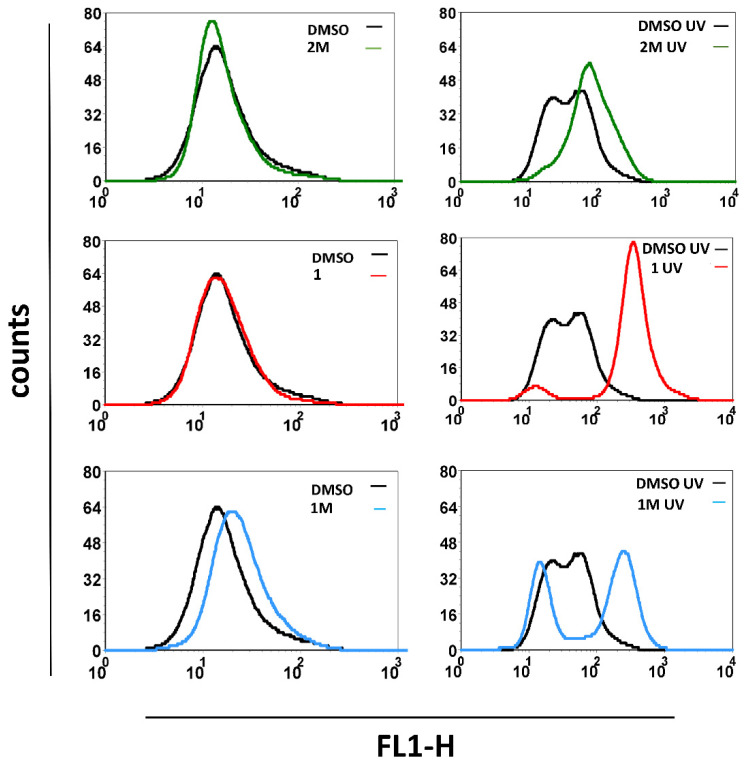
The amount of ROS in A549 cells treated with 10 µM **2M**, **1**, or **1M**, with or without UV light irradiation. Levels of ROS were assessed by measuring the fluorescence intensity of a DCF stain by flow cytometry. The black line represents control cells, i.e., cells treated with DMSO. Coloured lines represent cells treated with **2M**, **1**, or **1M**, respectively. Representative data from two independent experiments are shown, both yielding similar results.

Exposing human cells to pyrenes may result in increased production of reactive oxygen species (ROS) [6]. We, therefore, assessed the generation of ROS in A549 cells treated with 10 µM **2M**, **1**, or **1M**, under UV irradiation, while control experiments were kept in dark. As shown in Figure 8, treating cells with **1M** alone already induced the production of intracellular ROS.

The observed increase in ROS production was moderate, but distinctive. UV light irradiation of A549 cells treated with **2M**, **1**, and **1M** caused an immediate and strong increase in ROS production. A certain percentage of cells treated with **1M** disintegrated after UV exposure (see video experiment on a confocal microscope, Supplementary Information), and thus could not be stained by the DCF dye.

### 4.3. UV Light Irradiation Increases Cell Size of ***2M***, ***1*** or ***1M*** Treated A549 Cells

**Figure 9:**
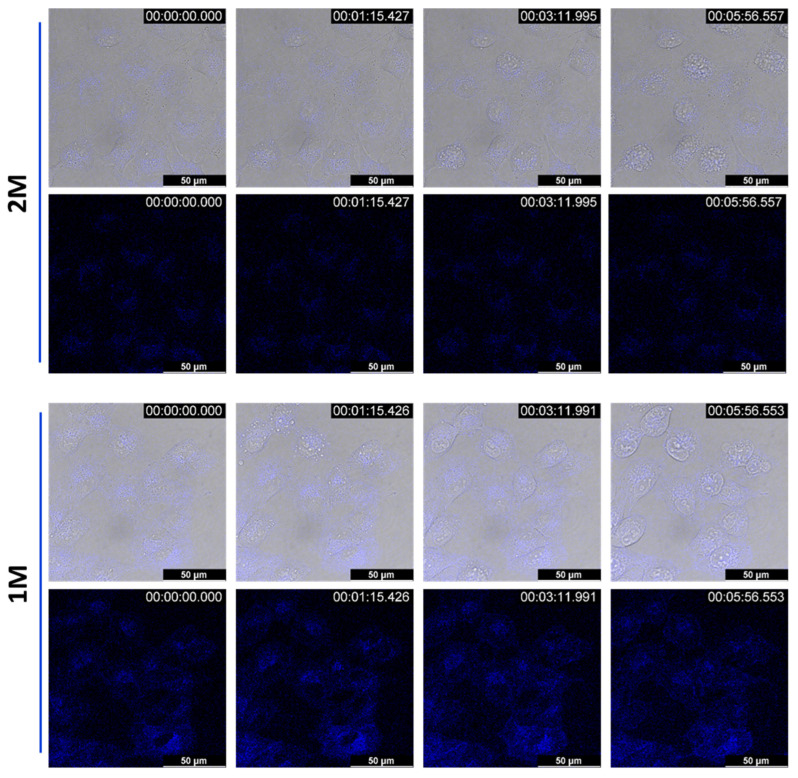
Confocal time-lapse imaging of A549 cells treated with **2M** or **1M** (1 µM) and irradiated at maximum diode power at λ_exc_ = 350 nm, monitored by bright field and fluorescence (λ_em_ = 420–500 nm) during 6 min. Note severe blebbing in the cells and complete disintegration after only 3 min irradiation, while non-treated cells did not change.

When observed on a confocal microscope, irradiated at 350 nm by a diode light source, **2M**- and **1M**-treated A549 cells exhibited swelling and even blebbing morphology (Figure 9), under continuous irradiation, already appearing within the first minute (see video experiment on a confocal microscope, Supplementary Information). In the control experiment, non-treated cells did not show any changes upon irradiation.

**Figure 10:**
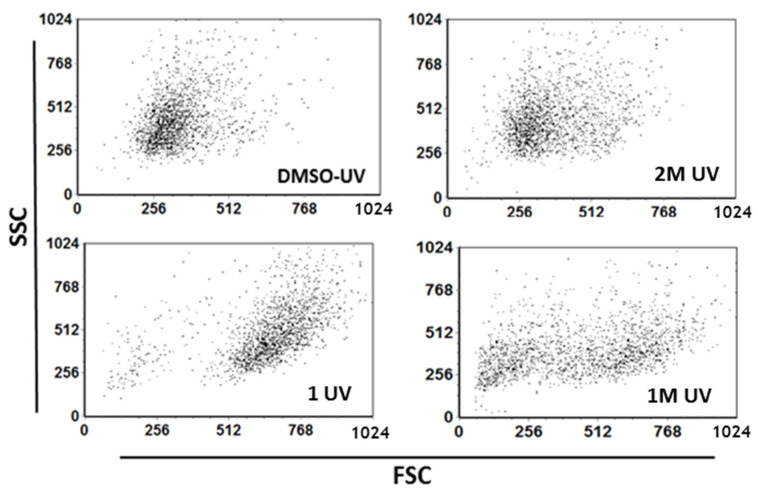
Exposing A549 cells treated with 10 µM **2M**, **1**, or **1M** to UV light induces changes in cell morphology. Cell size (FSC) and complexity (SSC) were assessed by flow cytometry. Representative data from two independent experiments are shown, both yielding similar results.

To corroborate further the observed phenomena, we determined morphology changes in **2M**-, **1**-, and **1M**-treated A549 cells after exposing them to UV light. Morphology changes were determined by flow cytometry, which allows the assessment of cell size (FSC) and complexity (SSC). UV irradiation caused an increase in cell size in **2M**-, **1**-, and **1M**-treated A549 cells as evidenced by an increase in FSC value (Figure 10). This increase was the most pronounced in cells treated with **1** and **1M**. Such morphological changes observed upon ROS production are consistent with the blebbing changes observed by confocal microscopy (see video experiment on a confocal microscope, Supplementary Information) and suggest strong singlet oxygen production induced by irradiation.

### 4.4. Intracellular Localisation of Compounds

**Figure 11:**
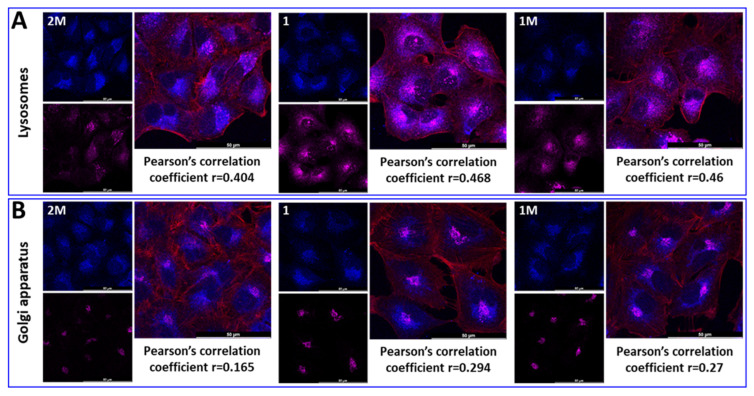
(**A**) Colocalisation of **2M**, **1**, and **1M** (blue, λ_exc_ = 350 nm; λ_em_ = 420–500 nm) with LAMP1 (magenta) observed by confocal microscopy. Cells were treated with 10 µM of the respective compound for 90 min at 37 °C, fixed with PFA, and subsequently stained with anti LAMP1 antibody. (**B**) Colocalisation of **2M**, **1**, and **1M** (blue) with GM130 (magenta) was observed by confocal microscopy. Cells were treated with 10 µM of the respective compound for 90 min at 37 °C, fixed with PFA, and subsequently stained with anti GM130 antibody. Colocalisation is assessed by the Pearson correlation coefficient. Analysis was conducted using ImageJ software and the appropriate JACoP plugin [53].

To investigate intracellular localisation of **2M**, **1**, and **1M** we performed co-localisation experiments with stains specific for various cytoplasmic organelles, namely mitochondria, lysosome, and Golgi apparatus (Figure 11).

Based on **2M**, **1**, and **1M** intracellular trafficking observed by live cell imaging, it was obvious that the compounds tested do not follow the pattern of early endosomes (Figure 7); however, we were not able to deduce whether some of them localise in lysosomes or Golgi. Thus, we treated cells with **2M**, **1**, and **1M**, fixed them with PFA and did colocalisation studies with LAMP1 as a marker for lysosomes and Gm130 as a marker for Golgi. All three compounds tested **2M**, **1**, and **1M**, show a modest degree of localisation in lysosomes (Pearson’s coefficients of 0.404, 0.468 and 0.46 respectively) (Figure 11A). Likewise, **2M**, **1**, and **1M** do not colocalise with Golgi (Pearson’s coefficients of 0.165, 0.294 and 0.27, respectively) (Figure 11B).

**Figure 12:**
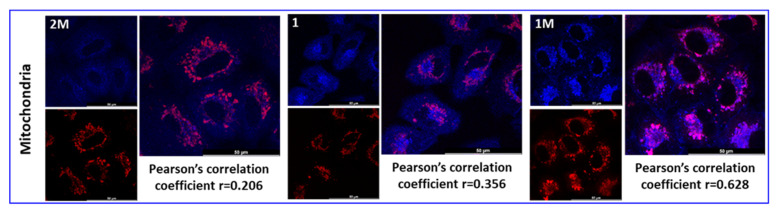
Colocalisation of **2M**, **1**, and **1M** (blue, λ_exc_ = 350 nm; λ_em_ = 420–500 nm) with MitoTracker^®^ (Deep Red) observed by confocal microscopy. Cells were treated with 10 µM of the compound for 90 min at 37 °C and incubated with 100 nM MitoTracker^®^ for 20 min at 37 °C. Collocalisation is assessed by Pearson’s coefficient. Analysis was conducted using ImageJ software and the appropriate JACoP plugin [53].

For assessing the colocalisation of **2M**, **1**, and **1M** with mitochondria, we used Mito Tracker Deep Red. As shown in Figure 12, **1M** shows significant colocalisation at mitochondria (Pearson’s coefficient of 0.628), while **2M** and **1** do not localize at mitochondria (Pearson’s coefficients of 0.206 and 0.356, respectively).

In conclusion, the compounds studied very efficiently enter living cells; however, none of them specifically accumulates at one intracellular organelle, and their intracellular distributions differ. Contrary to what would be expected from the observed strong DNA interactions (*vide supra*), the compounds do not bind to the cell nucleus. However, the monocation **1M** significantly accumulates in mitochondria, suggesting eventual interactions with mitoDNA. Dication **2M** and neutral compound **1** are non-selectively distributed over several different cellular organelles.

### 4.5. ***1M*** in Combination with UV Light Irradiation Acts on Mitochondria Causing Their Dysfunction by Increasing Their ROS Levels

As we saw that **1M** shows significant localisation at mitochondria, and having in mind its binding to DNA, as well as strong induction of total ROS production, we next investigated whether **1M** can cause mitochondrial damage. Therefore, we determined the production of mitochondrial ROS (mtROS) by quantification of MitoSOX Red intensities.

**Figure 13:**
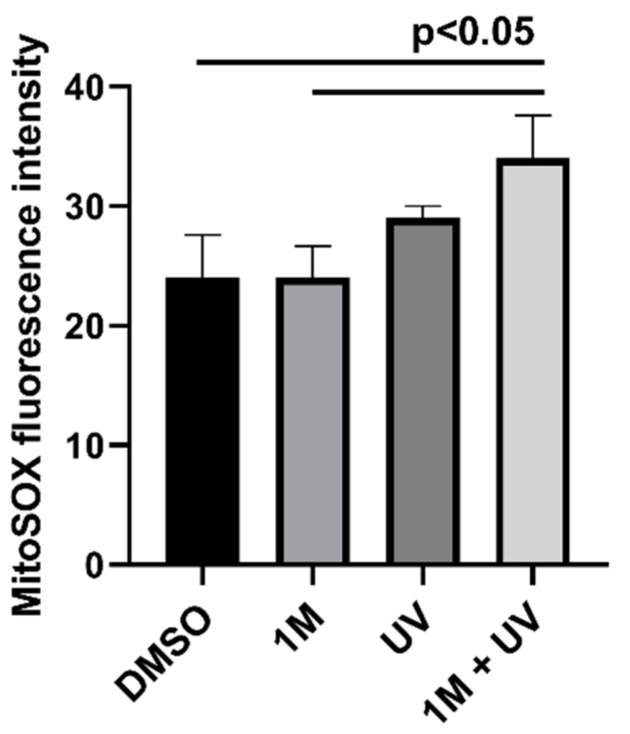
A combination of 1µM **1M** and UV light irradiation increases mtROS in A549 cells. The experiments were performed at least three times and representative data are shown. *p* < 0.05, **1M**+UV irradiation vs. DMSO and **1M**. Results are presented as mean ± SD.

We observed statistically significant differences in the production of mtROS between the groups studied, while combined treatment with **1M** and UV irradiation significantly increased mtROS levels compared to DMSO and **1M** alone (Figure 13). Our results strongly suggest that the combination of **1M** and UV light irradiation significantly and specifically increases mtROS levels, thus indicating that the combination of **1M** and UV may cause specific impairment of mitochondrial function by increasing mtROS levels.

## 5. Conclusions

In this study, we assessed in detail the interactions of previously synthesised, permanently positively charged compounds **1M** and **2M**, and their non-methylated analogues **1** and **2**, with important biological targets: DNA, RNA, and BSA. Permanently charged **1M** and **2M** showed high-affinity intercalation into ds-DNAs, accompanied by aggregation at an excess of dye over DNA/RNA.

The fluorescence from non-methylated compounds **1** and **2** was strongly dependent on the protonation of the pyridines and, as a result, they behave as fluorescence ratiometric probes for simultaneous detection of both ds-DNA and BSA at pH 5, relying on the ratio between intensities at 420 nm (BSA specific) and 520 nm (DNA specific). Even more useful was the possibility to switch-off ds-DNA sensing by adjustment to pH 7, at which only BSA yields a measurable emission change.

Due to the absence of DNA affinity at neutral pH, non-methylated compounds **1** and **2** did not cleave plasmid DNA upon irradiation, in contrast to the very efficient photoinduced DNA cleavage by permanently charged **1M** and **2M**. This photoinduced cleavage could be attributed to pyrene-mediated irradiation-induced production of singlet oxygen, which would cleave the DNA backbone close to the DNA-binding site of pyrene.

Further experiments in vitro on A549 and WI-38 cell lines revealed moderate cytotoxicities of **2M**, **1**, and **1M**, which increase strongly upon light irradiation. This effect is most pronounced for **1M**, yielding measurable photo-induced activity at submicromolar concentrations. Detailed experiments showed that light irradiation increased total ROS production in cells treated with **2M**, **1**, or **1M**, while **1M** induced ROS production even without UV irradiation. Besides ROS production, UV irradiation of **1**- and **1M**-treated cells also caused significant changes in cell size and morphology, namely cell swelling and augmentation of cellular complexity, all of which are markers for ROS-induced cellular damage.

The intracellular localisation of **2M**, **1**, and **1M** was very compound-dependent. The biologically most potent compound, **1M**, showed significant localisation at mitochondria, which led us to suspect that increased cell death observed in cells treated with this compound could be due to mitochondrial damage. Indeed, as we measured the production of mitochondrial ROS in UV-irradiated **1M**-treated cells, this showed that **1M**, in combination with UV light irradiation, specifically acts on mitochondria causing their dysfunction by increasing mitochondrial ROS levels. As **1M** induced plasmid DNA cleavage upon irradiation, we believe that increased mitochondrial ROS production could be caused by increased mitochondrial DNA damage. Namely, increased mitochondrial ROS production has been reported in human airway epithelial cells exposed to benzo(a)pyrene [60]. Similarly, exposure to benzo(a)pyrene has been shown to cause mitochondrial dysfunction and injury to neural cells [61].

Thus, compounds studied, owing to their fluorescence and bio-bioactivity, could be considered promising lead compounds for two-photon-excited theranostic applications [62].

## Figures and Tables

**Table 1:** Thermal denaturation experiments: ∆*T_m_*-values ^a^ (°C) for different ratios ^b^ ***r*** of pyrene conjugates added to the polynucleotide. See Supplementary Information for additional data.

	r	ct-DNA	Poly dAdT-Poly dAdT	pApU
**1M** ^d^	0.2	^c^ 2	8	3
	0.3	^c^ 2	9	3
**2M** ^d^	0.2	^d^ >16	31	4
	0.3	^d^ >16	33	-
**1** ^e^	0.1	0	-	-
	0.3	2	-	-
**2** ^e^	0.1	1.5	-	-

^a^ Error in ∆*T_m_*-values ± 0.5 °C. ^b^ r = [compound]/[polynucleotide].^c^ Published results [46]. ^d^ Conducted at pH 7.0, sodium cacodylate buffer*, I* = 50 mM. ^e^ Conducted at pH 5.0, sodium cacodylate buffer*, I* = 50 mM.

**Table 2:** Binding constants (^a^ log*K_s_*) of **1**, **2**, **1M** and **2M** with ds-polynucleotides and BSA at pH = 7.0 or pH = 5.0, sodium cacodylate buffer, *I* = 0.05 M.

		ct-DNA	BSA	GC-DNA	AU-RNA	AT-DNA
**1M**	pH 7	5.5	5.4	7.0	5.8	5.9
**2M**	pH 7	8.8	6.9	9.1	8.5	7.1
**1**	pH 7	b	5.5 5.5	-	-	-
	pH 5	5.3				
**2**	pH 7	b	5.3 5.1	-	-	-
	pH 5	5.7				

^a^ Processing of titration data by means of the Scatchard equation [56,57] gave values of ratio n[bound dye]/[polynucleotide] = 0.1 and 0.2. For easier comparison, all log*Ks* values were re-calculated for fixed n = 0.2 or n = 0.3. Correlation coefficients were > 0.99 for all calculated K_s_. ^b^ The changes were too small to allow an accurate calculation of the binding constant.

## Data Availability

Not applicable.

## Supplementary Materials

The following supporting information can be downloaded at: https://www.mdpi.com/article/10.3390/pharmaceutics14112499/s1, Figure S1: (a) Dependence of the UV/Vis spectrum of **1** (c = 2.0 × 10^−5^ M) on pH; (b,c) Dependence of the UV/Vis spectrum on pH at selected maxima. The p*K_a_* is 5; Figure S2: (a) Dependence of the UV/Vis spectrum of **2** (c = 2.0 × 10^−5^ M) on pH; (b) Dependence of the UV/Vis spectrum on pH at 318 m. The p*K_a_* is < 4; Figure S3: (a) Dependence of the fluorescence spectrum of **1** (c = 1.0 × 10^−6^ M; λ_exc_ = 340 nm) on pH; (b) Dependence of the fluorescence spectrum of **2** (c = 1.0 × 10^−6^ M; λ_exc_ = 332 nm) on pH; (c) Dependence of the UV/Vis spectrum on pH at 527 nm. The p*K_a_* is < 4; Figure S4: (a) Changes in the UV/vis spectrum of **1M** (c = 5.0 × 10^−6^ M) upon titration with ctDNA; (b) Dependence of the absorbance of **1M** at λ_max_ = 319 nm on c(ct-DNA), at pH 7.0, sodium cacodylate buffer, I = 0.05 M; Figure S5: (a) Changes in the UV/vis spectrum of **1M** (c = 5.0 × 10^−6^ M) upon titration with p(dAdT)_2_; (b) Dependence of the absorbance of **1M** at λ_max_ = 316 nm on *c*(p(dAdT)_2_), at pH 7.0, sodium cacodylate buffer, I = 0.05 M; Figure S6: (a) Changes in the UV/vis spectrum of **1M** (c = 5.0 × 10^−6^ M) upon titration with p(dGdC)_2_; (b) Dependence of the absorbance of **1M** at λ_max_ = 316 nm on *c*(p(dGdC)_2_), at pH 7.0, sodium cacodylate buffer, I = 0.05 M; Figure S7: (a) Changes in the UV/vis spectrum of **1M** (c = 5.0 × 10^−6^ M) upon titration with pApU; (b) Dependence of the absorbance of **1M** at λ_max_ = 316 nm on *c*(pApU), at pH 7.0, sodium cacodylate buffer, I = 0.05 M; Figure S8: (a) Changes in the UV/vis spectrum of **2M** (c = 5.0 × 10^−6^ M) upon titration with ct-DNA; (b) Dependence of the absorbance of **2M** at λ_max_ = 319 nm on *c*(ctDNA), at pH 7.0, sodium cacodylate buffer, I = 0.05 M; Figure S9: (a) Changes in the UV/vis spectrum of **2M** (c = 5.0 × 10^−6^ M) upon titration with p(dAdT)_2_; (b) Dependence of the absorbance of **2M** at λ_max_ = 319 nm on *c*(p(dAdT)_2_), at pH 7.0, sodium cacodylate buffer, I = 0.05 M; Figure S10: (a) Changes in the UV/vis spectrum of **2M** (c = 5.0 × 10^−6^ M) upon titration with p(dGdC)_2_; (b) Dependence of the absorbance of **2M** at λ_max_ = 319 nm on *c*(p(dGdC)_2_), at pH 7.0, sodium cacodylate buffer, I = 0.05 M; Figure S11: (a) Changes in the UV/VIS spectrum of **2M** (c = 5.0 × 10^−6^ M) upon titration with pApU; (b) Dependence of the absorbance of **2M** at λ_max_ = 319 nm on *c*(pApU), at pH 7.0, sodium cacodylate buffer, I = 0.05 M; Figure S12: (a) Changes in the fluorescence spectrum of **1** (c = 5.0 × 10^−7^ M) upon titration with ct-DNA at λ_exc_ = 340 nm; (b) Dependence of the intensity of the emission of **1** at λ_max_ = 540 nm on c(ct-DNA), at pH 5.0, sodium cacodylate buffer, I = 0.05 M; Figure S13: Changes in the fluorescence spectrum of **1** (c = 5.0 × 10^−7^ M) upon titration with BSA at λ_exc_ = 340 nm; sodium cacodylate buffer, I = 0.05 M: (a) pH 7.0 (b) pH 5.0; Figure S14: (a) Changes in the fluorescence spectrum of 2 (c = 5.0 × 10^−7^ M) upon titration with BSA at λ_exc_ = 319 nm; (b) Dependence of the intensity of the emission of 2 at λ_max_ = 418 nm on c(BSA), at pH 7.0, sodium cacodylate buffer, I = 0.05 M; Figure S15: (a) Changes in the fluorescence spectrum of **1M** (c = 5.0 × 10^−7^ M, λ_exc_ = 336 nm) upon titration with BSA; (b) Dependence of the intensity of the emission of 1M at λ_max_ = 476 nm on c(BSA), at pH 7.0, sodium cacodylate buffer, I = 0.05 M; Figure S16: (a) Changes in the fluorescence spectrum of **2M** (c = 5.0 × 10^−7^ M, λ_exc_ = 336 nm) upon titration with BSA; (b) Dependence of the intensity of the emission of **2M** at λ_max_ = 526 nm on c(BSA), at pH 7.0, sodium cacodylate buffer, I = 0.05 M; Figure S17: (a) Melting curve of ctDNA upon addition r = 0.2 and r = 0.3 ([compound/[polynucleotide]) of **1M** at pH 7.0 (buffer sodium cacodylate, I = 0.05 M), b) first derivative of the absorbance vs. temperature; Figure S18: (a) Melting curve of p(dAdT)_2_ upon addition r = 0.2 and r = 0.3 ([compound]/[polynucleotide]) of 1M at pH 7.0 (buffer sodium cacodylate, I = 0.05 M), (b) first derivative of the absorbance vs. temperature; Figure S19: (a) Melting curve of pApU upon addition r = 0.2 and r = 0.3 ([compound]/[polynucleotide]) of **1M** at pH 7.0 (buffer sodium cacodylate, I = 0.05 M), (b) first derivative of the absorbance vs. temperature; Figure S20: (a) Melting curve of ctDNA upon addition r = 0.2 and r = 0.3 ([compound]/[polynucleotide]) of **2M** at pH 7.0 (buffer sodium cacodylate, I = 0.05 M), (b) first derivative of the absorbance vs. temperature; Figure S21: (a) Melting curve of p(dAdT)_2_ upon addition r = 0.2 and r = 0.3 ([compound]/[polynucleotide]) of **2M** at pH 7.0 (buffer sodium cacodylate, I = 0.05 M), (b) first derivative of the absorbance vs. temperature; Figure S22: (a) Melting curve of pApU upon addition r = 0.2 and r = 0.3 ([compound]/[polynucleotide]) of **2M** at pH 7.0 (buffer sodium cacodylate, I = 0.05 M), (b) first derivative of the absorbance vs. temperature; Figure S23: CD titration of poly dGdC - poly dGdC (c = 2 × 10^−5^ M) with **1M** at molar ratios r = [compound]/[polynucleotide] (pH 7.0, buffer sodium cacodylate, I = 0.05 M); Figure S24: CD titration of ct-DNA, (c = 2 × 10^−5^ M) with (a) 1 (b) 2, at molar ratios r[compound]/[DNA] = 0.1–0.3. Done at pH 5.0, buffer sodium cacodylate, I = 0.05 M; Figure S25: Cell survival of A549 cells exposed to compound 2. Data from 4 replicates are presented as mean ± SD, relative to the control samples. Control samples are cells treated with DMSO at the same concentration as the tested compound. Representative data from three independent experiments, which yielded similar results, are shown; Table S1: Groove widths and depths for selected nucleic acid conformations. References [63,64] are cited in the Supplementary Materials.
