# Supplementary material for: Para-N-Methylpyridinium Pyrenes: Impact of Positive Charge on ds-DNA/RNA and Protein Recognition, Photo-Induced Bioactivity, and Intracellular Localisation
Source: Pharmaceutics. 2022 Nov 17;14(11):2499. doi: 10.3390/pharmaceutics14112499 (PMC9696974; doi:10.3390/pharmaceutics14112499)
Supplement: Supplementary file 1 [file pharmaceutics-14-02499-s001.zip › pharmaceutics-2011810-supplementary.pdf]

Supporting Information

***Para*-N-Methylpyridinium Pyrenes: Photodynamic Bioactivity Mechanism and Intracellular Distribution Dependence on Cationic Substituents**

Table of Contents

1. Dependence of UV/Vis and fluorescence spectra of **1** and **2** on pH 2

2. Study of interactions with double-stranded DNA/RNA 4

2.1. Structural properties of DNA and RNA 4

2.2. UV/VIS spectrophotometric titrations 4

2.3. Fluorimetric titrations 7

2.4. Thermal melting experiments 10

2.5. CD experiments: 12

3. Biological results 13

4. Synthetic procedures 13

1. Dependence of UV/Vis and fluorescence spectra of **1** and **2** on pH

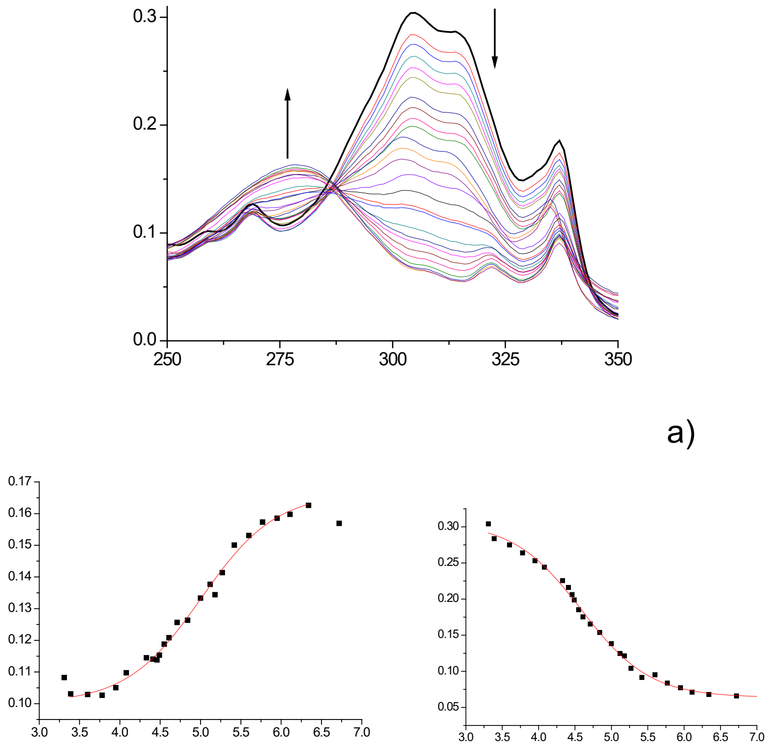

a)

b) c)

Figure S1. a) Dependence of the UV/Vis spectrum of **1** (c = 2.0 × 10^{-5} M) on pH; b,c) Dependence of the UV/Vis spectrum on pH at selected maxima. The p*Ka* is 5.

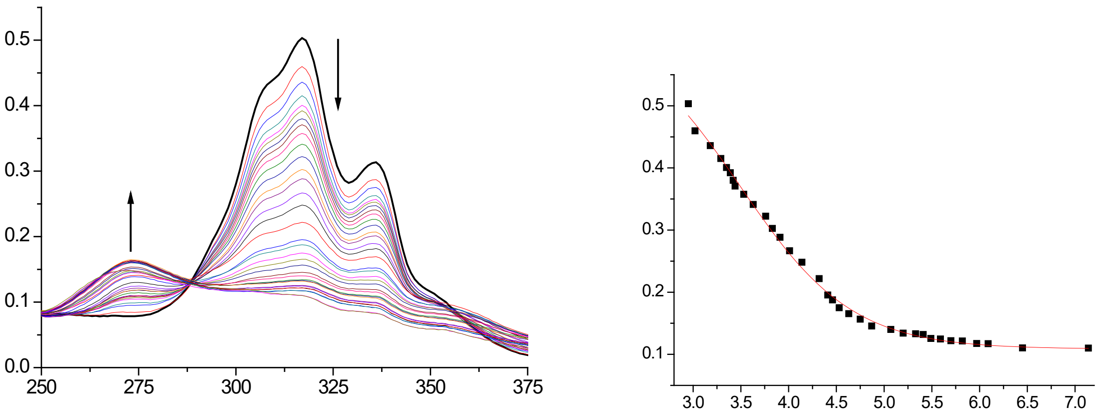

a) b)

Figure S2. a) Dependence of the UV/Vis spectrum of **2** (c = 2.0 × 10^{-5} M) on pH; b) Dependence of the UV/Vis spectrum on pH at 318 m. The p*Ka* is < 4.

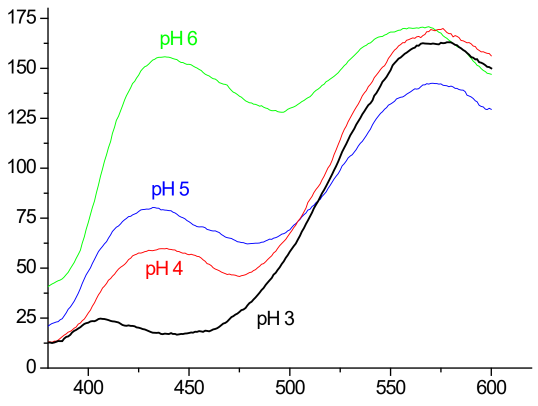

a)

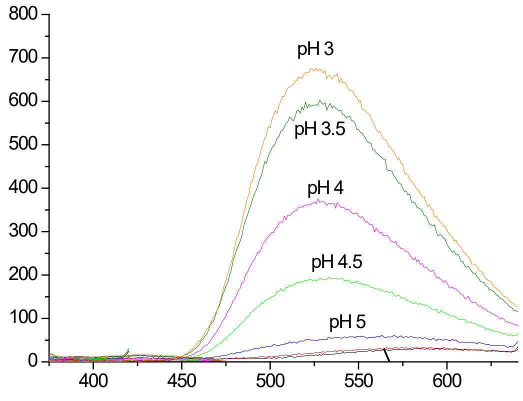

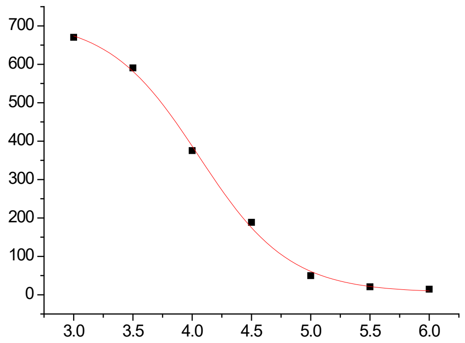

b) c)

Figure S3. a) Dependence of the fluorescence spectrum of **1** (c = 1.0 × 10^{-6} M; λ_{exc} = 340 nm) on pH; b) Dependence of the fluorescence spectrum of **2** (c = 1.0 × 10^{-6} M; λ_{exc} = 332 nm) on pH; c) Dependence of the UV/Vis spectrum on pH at 527 nm. The p*Ka* is < 4.

2. Study of interactions with double-stranded DNA/RNA

2.1. Structural properties of DNA and RNA

Table S1. Groove widths and depths for selected nucleic acid conformations [1].

Structure type	Groove width [Å]		Groove depth [Å]	
	major	minor	major	minor
[a] poly rA – poly rU	3.8	10.9	13.5	2.8
[b] ct-DNA (48% of GC-pairs)	11.4	3.3	7.5	7.9
[b] poly dAdT – poly dAdT	11.2	6.3	8.5	7.5
[c] poly dGdC – poly dGdC	13.5	9.5	10.0	7.2

[a] A - helical structure

[b] B - helical structure

[c] B- helical structure with sterically blocked minor groove by amino groups of guanines

2.2. UV/VIS spectrophotometric titrations

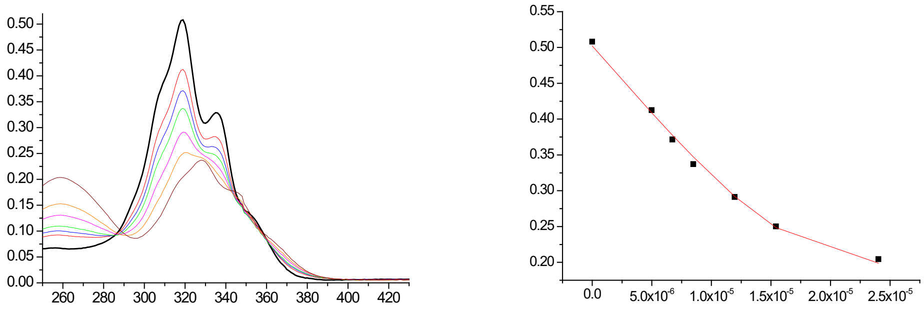

a) b)

Figure S4. a) Changes in the UV/vis spectrum of **1M** (c = 5.0 × 10^{-6} M) upon titration with ctDNA; **b)** Dependence of the absorbance of **1M** at λ_{max} = 319 nm on c(ct-DNA), at pH 7.0, sodium cacodylate buffer, I = 0.05 M.

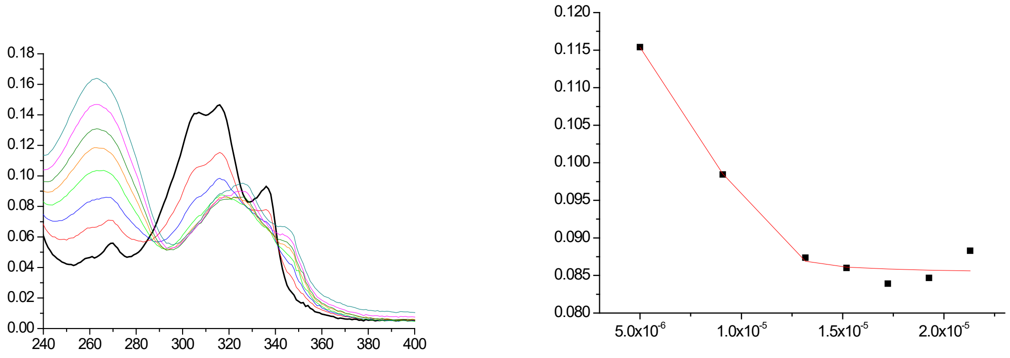

a) b)

Figure S5. a) Changes in the UV/vis spectrum of **1M** (c = 5.0 × 10^{-6} M) upon titration with p(dAdT)$_2$; **b)** Dependence of the absorbance of **1M** at λ_{max} = 316 nm on c(p(dAdT)$_2$), at pH 7.0, sodium cacodylate buffer, I = 0.05 M.

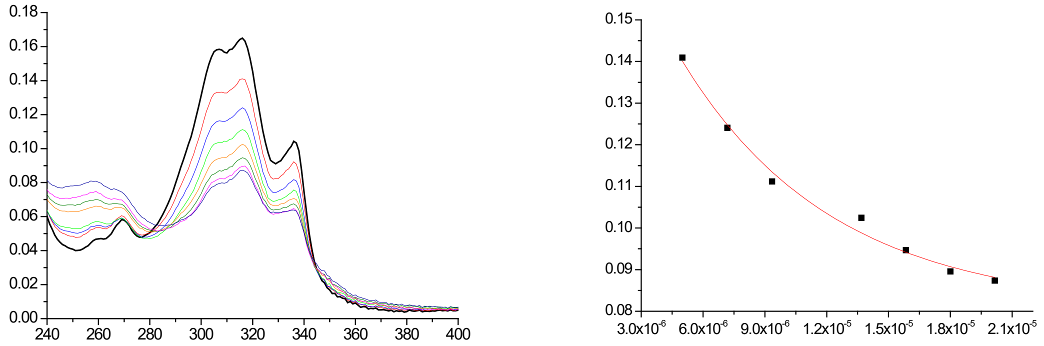

a) b)

Figure S6. a) Changes in the UV/vis spectrum of **1M** (c = 5.0 × 10^{-6} M) upon titration with p(dGdC)$_2$; **b)** Dependence of the absorbance of **1M** at λ_{max} = 316 nm on c(p(dGdC)$_2$), at pH 7.0, sodium cacodylate buffer, I = 0.05 M.

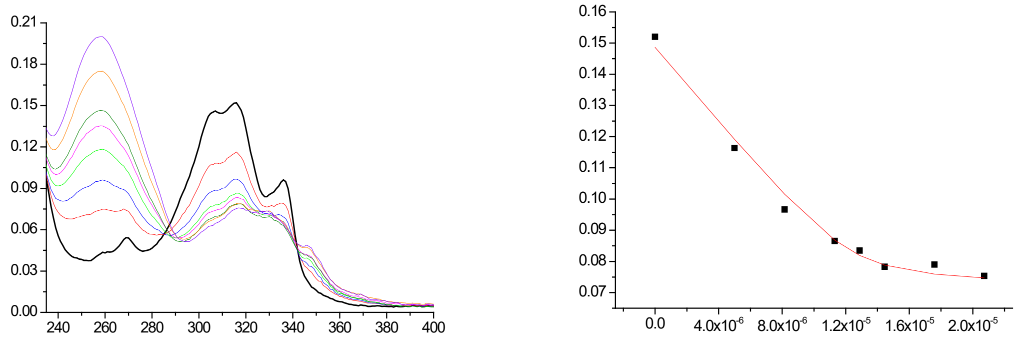

a) b)

Figure S7. a) Changes in the UV/vis spectrum of **1M** (c = 5.0 × 10^{-6} M) upon titration with pApU; **b)** Dependence of the absorbance of **1M** at λ_{max} = 316 nm on c(pApU), at pH 7.0, sodium cacodylate buffer, I = 0.05 M.

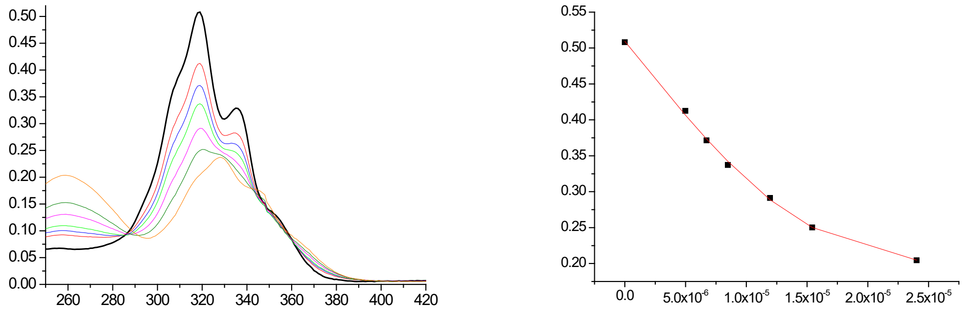

a) b)

Figure S8. a) Changes in the UV/vis spectrum of **2M** (c = 5.0 × 10^{-6} M) upon titration with ct-DNA; **b)** Dependence of the absorbance of **2M** at λ_{max} = 319 nm on c(ctDNA), at pH 7.0, sodium cacodylate buffer, I = 0.05 M.

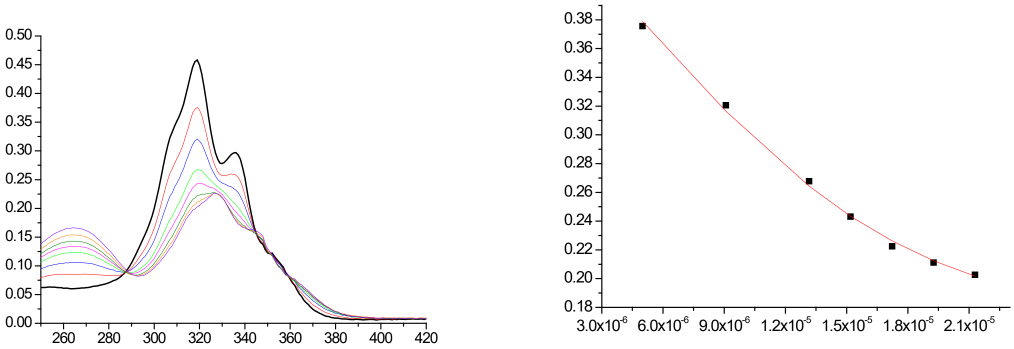

a) b)

Figure S9. a) Changes in the UV/vis spectrum of **2M** (c = 5.0 × 10^{-6} M) upon titration with p(dAdT)$_2$; **b)** Dependence of the absorbance of **2M** at λ_{max} = 319 nm on c(p(dAdT)$_2$), at pH 7.0, sodium cacodylate buffer, I = 0.05 M.

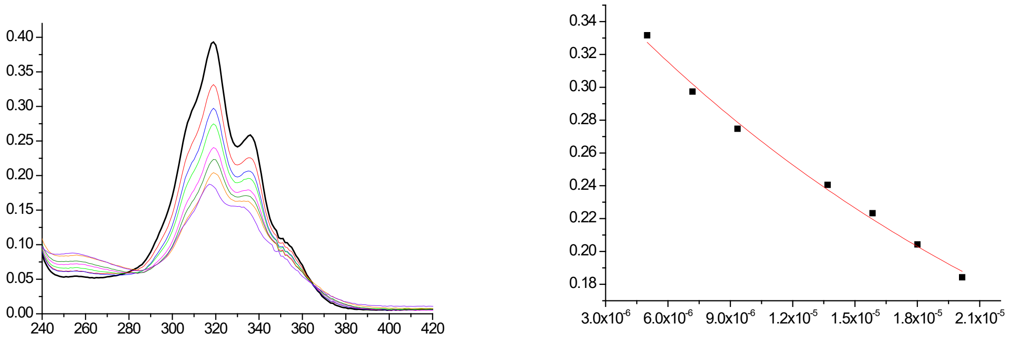

a) b)

Figure S10. a) Changes in the UV/vis spectrum of **2M** (c = 5.0 × 10^{-6} M) upon titration with p(dGdC)$_2$; **b)** Dependence of the absorbance of **2M** at λ_{max} = 319 nm on c(p(dGdC)$_2$), at pH 7.0, sodium cacodylate buffer, I = 0.05 M.

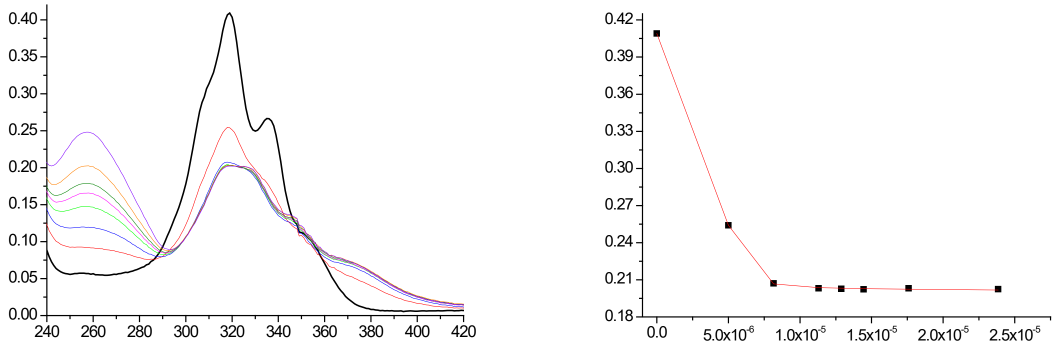

a) b)

Figure S11. a) Changes in the UV/VIS spectrum of **2M** (c = 5.0 × 10^{-6} M) upon titration with pApU; **b)** Dependence of the absorbance of **2M** at λ_{max} = 319 nm on c(pApU), at pH 7.0, sodium cacodylate buffer, I = 0.05 M.

2.3. Fluorimetric titrations

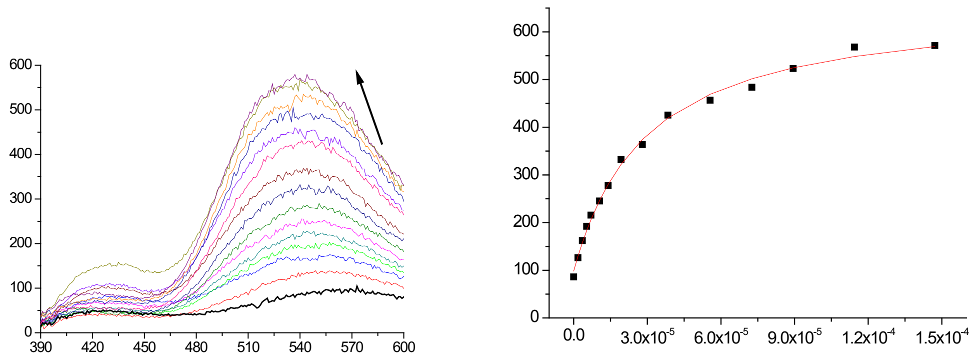

a) b)

Figure S12. a) Changes in the fluorescence spectrum of **1** (c = 5.0 × 10^{-7} M) upon titration with ct-DNA at λ_{exc} = 340 nm; **b)** Dependence of the intensity of the emission of **1** at λ_{max} = 540 nm on c(ct-DNA), at pH 5.0, sodium cacodylate buffer, I = 0.05 M.

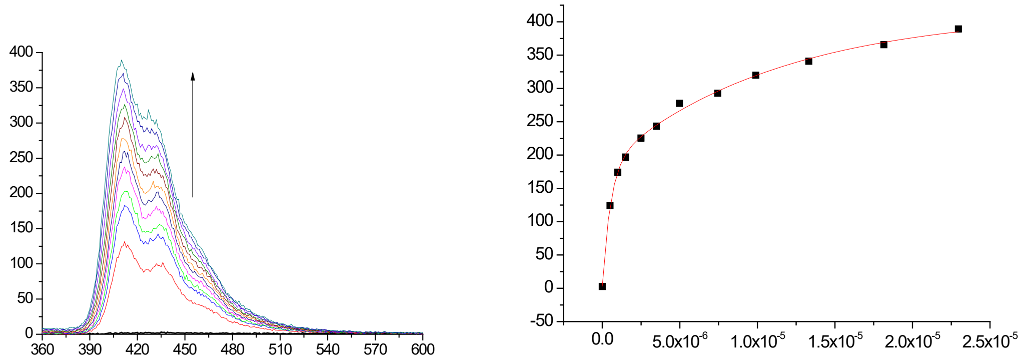

a)pH7

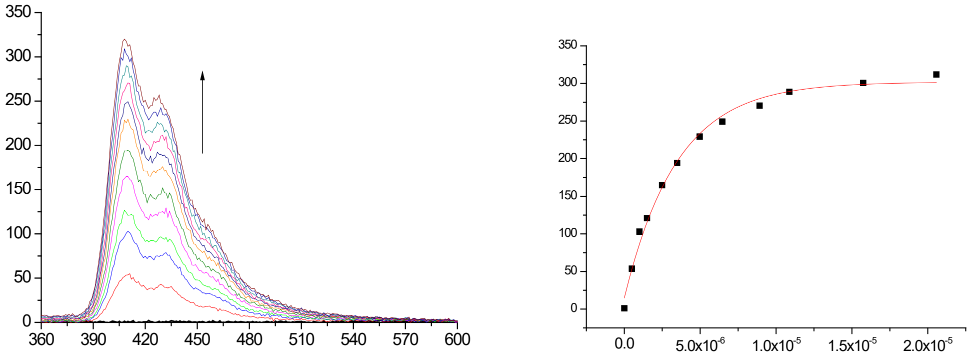

b)pH5

Figure S13. Changes in the fluorescence spectrum of **1** (c = 5.0 × 10^{-7} M) upon titration with BSA at λ_{exc} = 340 nm; sodium cacodylate buffer, I = 0.05 M: a) pH 7.0 b) pH 5.0.

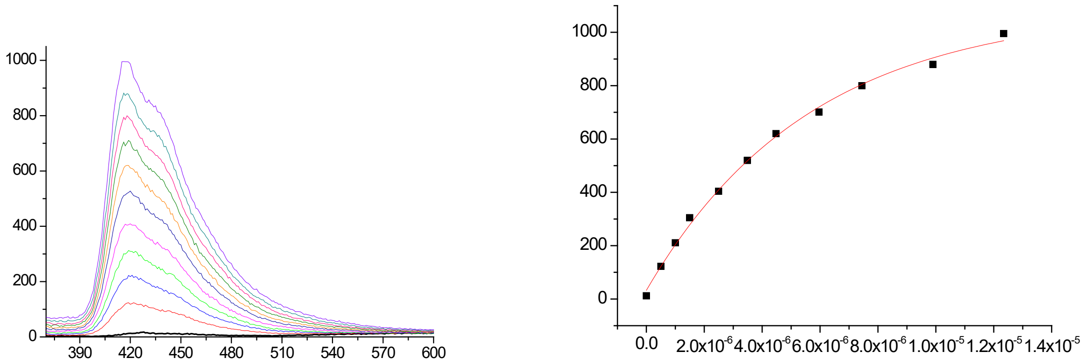

a) b)

Figure S14. a) Changes in the fluorescence spectrum of **2** (c = 5.0 × 10^{-7} M) upon titration with BSA at λ_{exc} = 319 nm; b) Dependence of the intensity of the emission of **2** at λ_{max} = 418 nm on c(BSA), at pH 7.0, sodium cacodylate buffer, I = 0.05 M.

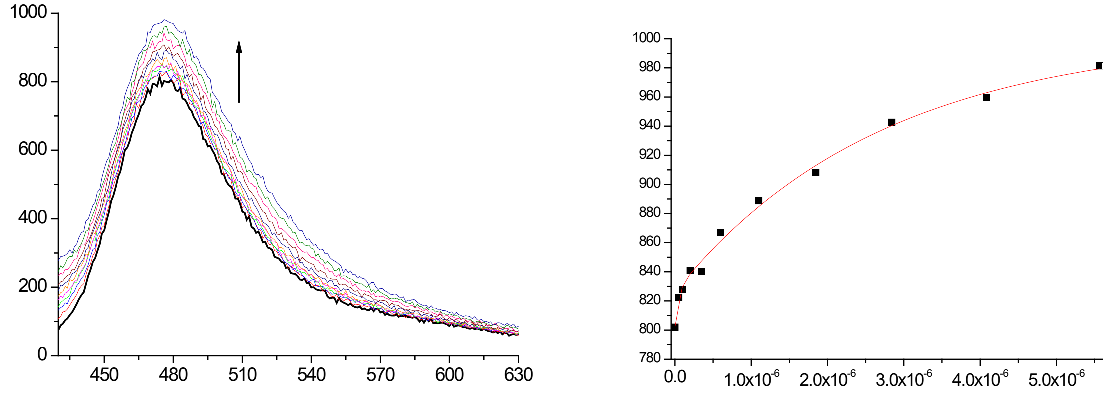

a) b)

Figure S15. a) Changes in the fluorescence spectrum of **1M** (c = 5 ×.0 10^{-7} M, λ_{exc} = 336 nm) upon titration with BSA; b) Dependence of the intensity of the emission of **1M** at λ_{max} = 476 nm on c(BSA), at pH 7.0, sodium cacodylate buffer, I = 0.05 M.

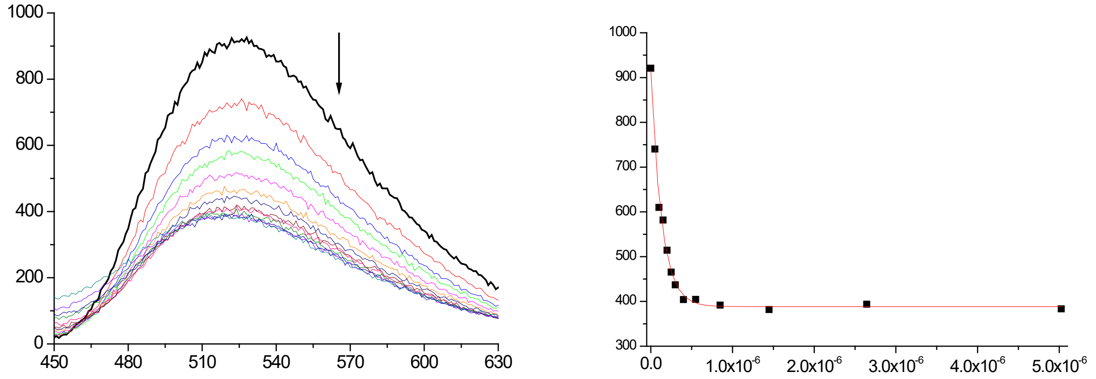

a) b)

Figure S16. a) Changes in the fluorescence spectrum of **2M** (c = 5.0 × 10^{-7} M, λ_{exc} = 336 nm) upon titration with BSA; b) Dependence of the intensity of the emission of **2M** at λ_{max} = 526 nm on c(BSA), at pH 7.0, sodium cacodylate buffer, I = 0.05 M.

2.4. Thermal melting experiments

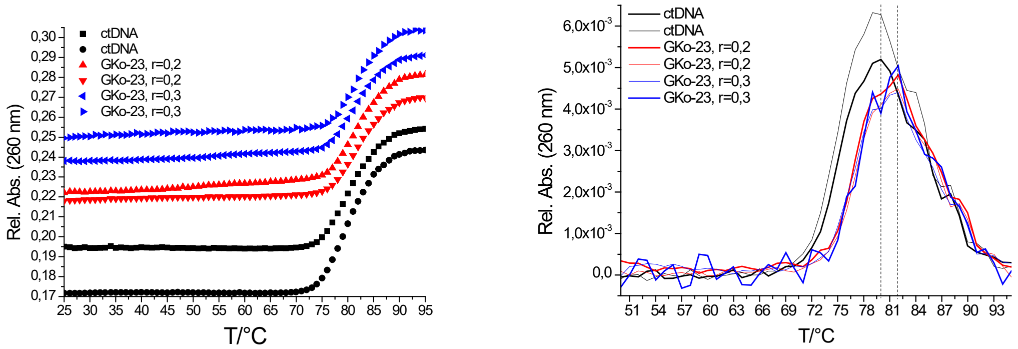

a) b)

Figure S17. a) Melting curve of ctDNA upon addition r = 0.2 and r = 0.3 ([compound/ [polynucleotide]) of **1M** at pH 7.0 (buffer sodium cacodylate, *I* = 0.05 M), **b)** first derivative of the absorbance vs. temperature.

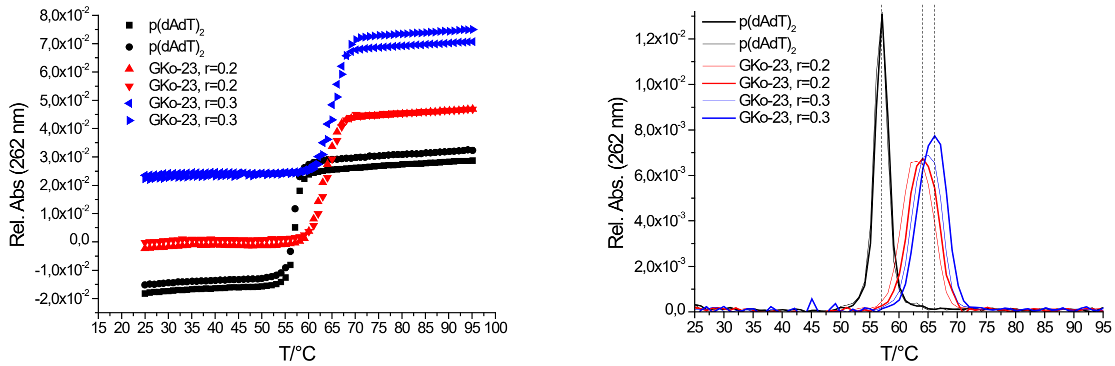

a) b)

Figure S18. a) Melting curve of p(dAdT)$_2$ upon addition r = 0.2 and r = 0.3 ([compound]/ [polynucleotide]) of **1M** at pH 7.0 (buffer sodium cacodylate, *I* = 0.05 M), **b)** first derivative of the absorbance vs. temperature.

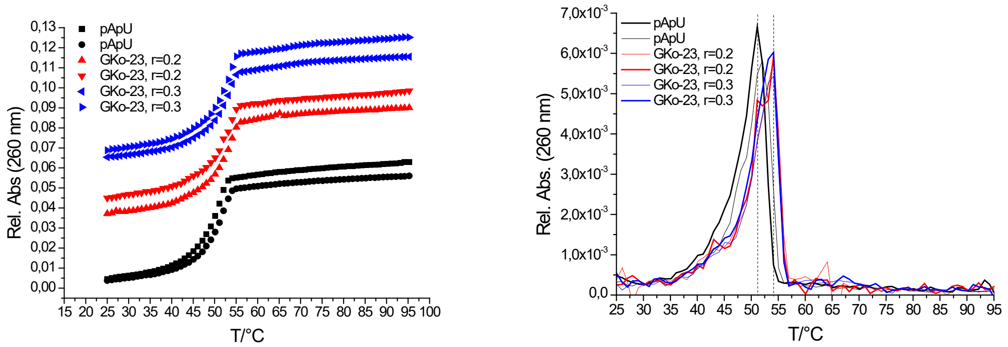

a) b)

Figure S19. a) Melting curve of pApU upon addition r = 0.2 and r = 0.3 ([compound]/ [polynucleotide]) of **1M** at pH 7.0 (buffer sodium cacodylate, *I* = 0.05 M), **b)** first derivative of the absorbance vs. temperature.

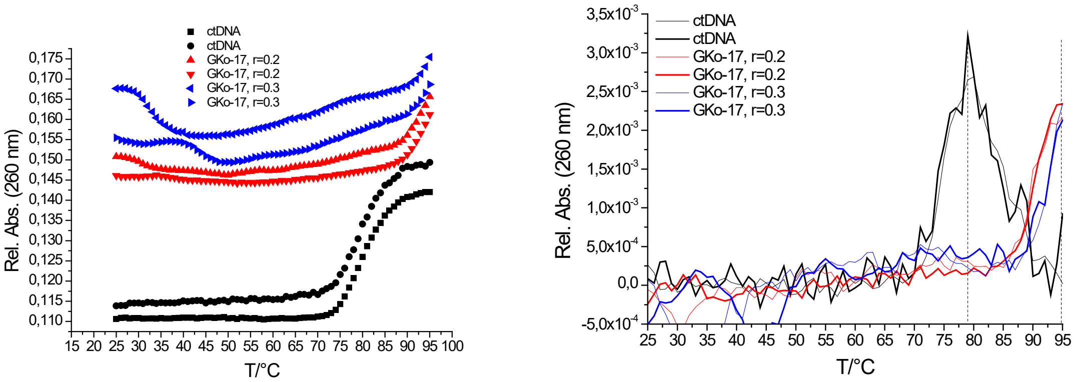

a) b)

Figure S20. a) Melting curve of ctDNA upon addition r = 0.2 and r = 0.3 ([compound]/[polynucleotide]) of **2M** at pH 7.0 (buffer sodium cacodylate, *I* = 0.05 M), **b)** first derivative of the absorbance vs. temperature.

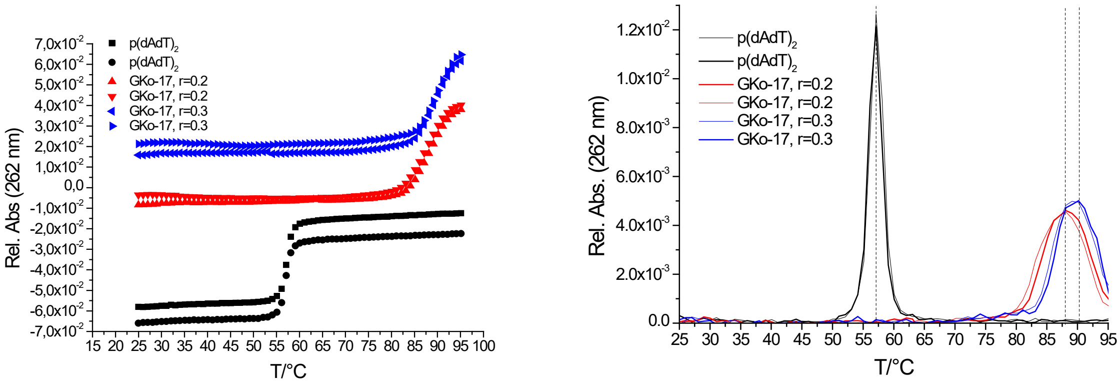

a) b)

Figure S21. a) Melting curve of p(dAdT)$_2$ upon addition r = 0.2 and r = 0.3 ([compound]/[polynucleotide]) of **2M** at pH 7.0 (buffer sodium cacodylate, *I* = 0.05 M), **b)** first derivative of the absorbance vs. temperature.

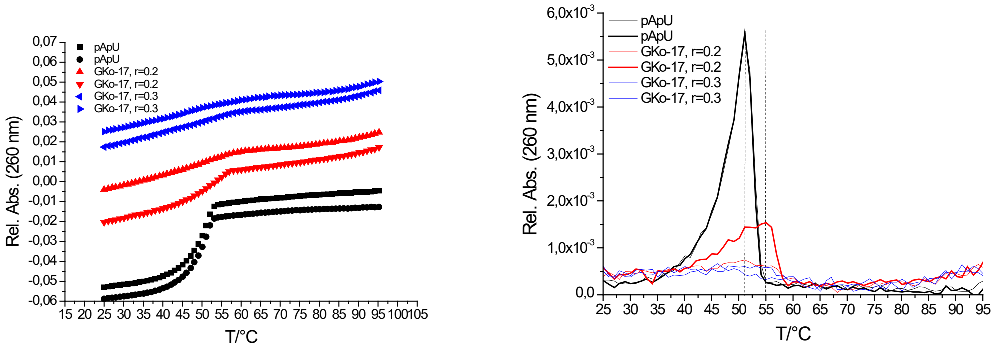

a) b)

Figure S22. a) Melting curve of pApU upon addition r = 0.2 and r = 0.3 ([compound]/[polynucleotide]) of **2M** at pH 7.0 (buffer sodium cacodylate, *I* = 0.05 M), **b)** first derivative of the absorbance vs. temperature.

2.5. CD experiments:

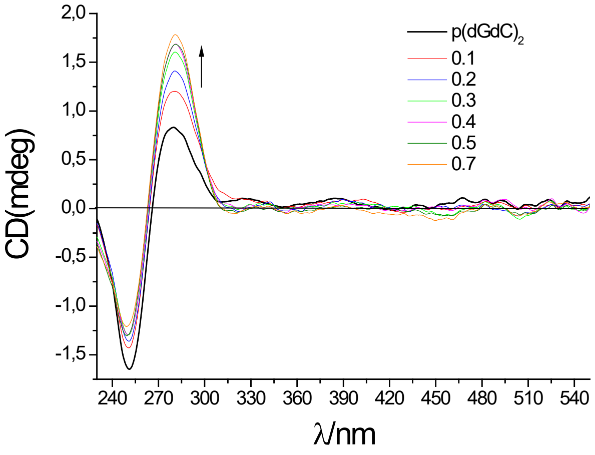

Figure S23. CD titration of poly dGdC - poly dGdC (c = 2 × 10^{-5} M) with **1M** at molar ratios r = [compound] / [polynucleotide] (pH 7.0, buffer sodium cacodylate, I = 0.05 M).

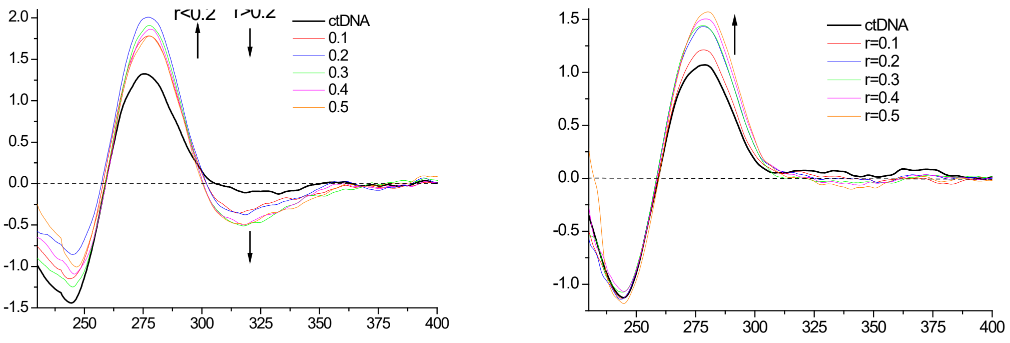

a) b)

Figure S24. CD titration of ct-DNA, (c = 2 × 10^{-5} M) with a) **1** b) **2**, at molar ratios r[compound] / [DNA] = 0.1-0.3. Done at pH 5.0, buffer sodium cacodylate, *I* = 0.05 M.

3. Biological results

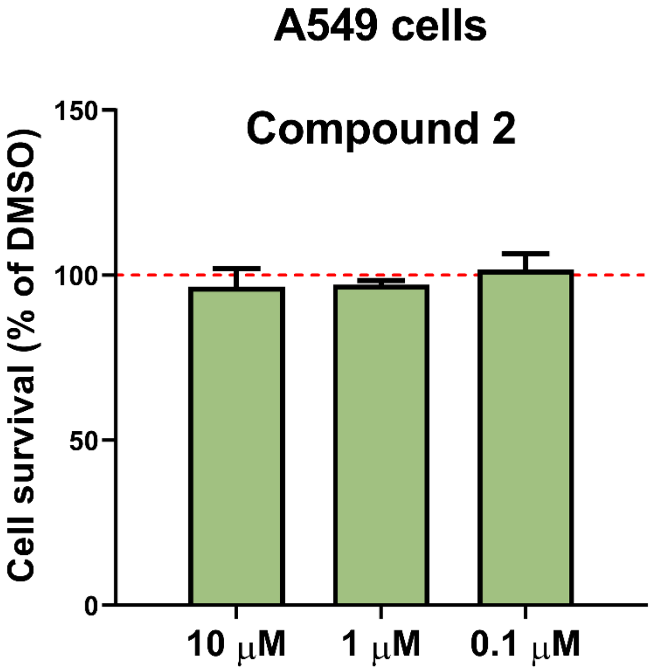

Figure S25. Cell survival of A549 cells exposed to compound **2**. Data from 4 replicates are presented as mean ± SD, relative to the control samples. Control samples are cells treated with DMSO at the same concentration as the tested compound. Representative data from three independent experiments, which yielded similar results, are shown.

4. Synthetic procedures

As described in the work published previously [2]:

Synthesis of 1: A mixture of 4-ethynylpyridine (0.60 g, 5.82 mmol), 1,4-diiodobenzene (0.85 g, 2.58 mmol), [Pd(dppf)Cl2] (0.11 g, 0.15 mmol) and CuI (0.03 g, 0.16 mmol) was added to dried and deoxygenated triethylamine (150 mL) under an argon atmosphere. The reaction mixture was stirred for 24 h after which the solvent was removed in vacuo. The solid residue was extracted with CH2Cl2 and the resulting solution was eluted through a short column of alumina (4 cm). Removal of the solvent gave the desired compound as a white powder. Yield 0.60 g (83%). 1H NMR (300 MHz, CDCl3): δ 8.63 (dd, J1 = 5 Hz, J2 = 2 Hz, 4H), 7.57 (s, 4H), 7.41 (dd, J1 = 5 Hz, J2 = 2 Hz, 4H). 13C{1H} NMR: δ 149.8, 131.9, 131.1, 125.5, 122.9, 93.3, 88.8. HRMS (ASAP+): M/Z found = 281.1063; M/Z calculated for [M+H]+ (C20H13N2+) = 281.1073.

Synthesis of 1M: Compound 1 (0.14 g, 0.50 mmol) was stirred with methyl triflate (160 μL, 0.24 g, 1.46 mmol) in dry toluene (15 ml) over 24 h in an argon atmosphere. The yellow precipitate obtained was isolated by filtration, washed with dry toluene and dried. Yield: 0.25 g (82%). Thin plate-like single crystals were obtained by slowly

evaporating a MeCN solution. 1H NMR (300 MHz, CD3CN): δ 8.58 (d, J = 7 Hz, 4H), 8.02 (d, J = 7 Hz, 4H), 7.78 (s, 4H), 4.26 (s, 6H). HRMS (ESI+): M/Z found = 155.0727; M/Z calculated for [M−2(OTf)]2+ (C22H18N22+) = 155.0730. Elem. Anal. Calcd. (%) C24H18F6N2O6S2: C 47.37, H 2.98, N 4.60, S 10.54: found: C 46.78, H 3.34, N 4.70, S 10.78.

Synthesis of 2: A mixture of 4-ethynylpyridine (0.60 g, 5.82 mmol), 1,4-diiodotetrafluorodobenzene (1.00 g, 2.49 mmol), [Pd(dppf)Cl2] (0.11 g, 0.15 mmol) and CuI (0.03 g, 0.16 mmol) was added to dried and deoxygenated triethylamine (150 mL) under an argon atmosphere. The reaction mixture was stirred for 36 h after which the solvent was removed in vacuo. The solid residue was extracted with CH2Cl2 and the resulting solution was eluted through a short column of alumina (4 cm). Removal of the solvent gave the desired compound as a brown powder. Yield 0.62 g (70%). 1H NMR (300 MHz, CDCl3): δ 8.69 (dd, J1 = 5 Hz, J2 = 2 Hz, 4H), 7.46 (dd, J1 = 5 Hz, J2 = 2 Hz, 4H). 19F{1H} NMR: δ -135.6. HRMS (ASAP+): M/Z found = 353.0697; M/Z calculated for [M+H]+ (C20H9F4N2+) = 353.0696. Elem. Anal. Calcd. (%) C20H8F4N2: C 68.19, H 2.29, N 7.95; found: C 67.72, H 2.37, N 8.10.

Synthesis of 2M: Compound 2 (0.18 g, 0.51 mmol) was stirred with methyl triflate (160 μl, 0.24 g, 1.46 mmol) in dry toluene (15 ml) over 24 h in an argon atmosphere. The light brown precipitate obtained was isolated by filtration, washed with dry toluene and dried. Yield: 0.29 g (85%). Needle like single crystals were obtained by slowly evaporating a MeCN solution. 1H NMR (300 MHz, CD3CN): δ 8.68 (d, J = 7 Hz, 4H), 8.13 (d, J = 7 Hz, 4H), 4.31 (s, 6H). 19F{1H} NMR (470 MHz, CD3CN): δ: −79.3 (OTf), −135.6. HRMS (ESI+): M/Z found: 191.0538; M/Z calculated for [M−2(OTf)]2+ (C22H14F4N22+) = 191.0541. Elem. Anal. Calcd. (%) C24H14F10N2O6S2: C 42.36, H 2.07, N 4.12, S 9.42: found: C41.63, H 2.29, N 3.73, N 9.15.

References:

[1] a) Saenger, W. *Principles of Nucleic Acid Structure*; *Springer-Verlag* **1983,** New York; b) Cantor, C. R.; Schimmel; P. R. Biophysical Chemistry. *WH Freeman and Co.* **1980**, *3*, 1109-1181, San Francisco.

[2] Kole, G.K.; Merz, J.; Amar, A.; Fontaine, B.; Boucekkine, A.; Nitsch, J.; Lorenzen, S.; Friedrich, A.; Krummenacher, I.; Košćak, M.; Braunschweig, H.; Piantanida, I.; Halet, J.-F.; Müller-Buschbaum, K.; Marder, T.B. 2- and 2,7-Substituted para-N-Methylpyridinium Pyrenes: Syntheses, Molecular and Electronic Structures, Photophysical, Electrochemical, and Spectroelectrochemical Properties and Binding to Double-Stranded (ds) DNA. *Eur. J. Chem.* **2021**, *27*, 2837-2853.
